# Quantum Chemistry-Driven Molecular Inverse Design of Stable Isomers with Data-Free Reinforcement Learning

**DOI:** 10.1021/acs.jctc.5c02055

**Published:** 2026-03-16

**Authors:** Francesco Calcagno, Luca Serfilippi, Giorgio Franceschelli, Marco Garavelli, Mirco Musolesi, Ivan Rivalta

**Affiliations:** † Department of Industrial Chemistry, 9296 Alma Mater Studiorum University of Bologna, Bologna 40129, Italy; ‡ Center for Chemical Catalysis - C3, Alma Mater Studiorum University of Bologna, Bologna 40129, Italy; § Department of Computer Science and Engineering, Alma Mater Studiorum University of Bologna, Bologna 40136, Italy; ∥ Department of Computer Science, 4919 University College London, London WC1E 6BT, U.K.

## Abstract

The inverse design (ID) of molecules remains one of the greatest challenges in chemistry. Machine learning and artificial intelligence (AI) methods are increasingly employed to generate candidate molecules with tailored properties but mostly rely on pretraining over large data sets, which introduces bias. Here, we present a data-free generative AI model called PROTEUS that integrates reinforcement learning with on-the-fly quantum mechanical calculations to enable the *de novo* design of molecules from first-principles. The AI tool uses a custom syntax and hierarchical learning architecture to navigate the chemical space without prior knowledge, optimizing the desired chemical property. We demonstrate the efficiency of our software by solving complex molecular design tasks related to the maximization of isomerization energy gaps for styrene derivatives. By solving ID problems for which the exact solutions are known, PROTEUS proved to be robust and flexible enough to perform a broad exploration of different chemical spaces while successfully exploiting chemical rewards. This framework opens new avenues for quantum chemistry-driven unbiased molecular design, offering a flexible and scalable strategy to address design challenges in chemistry.

## Graphical abstract

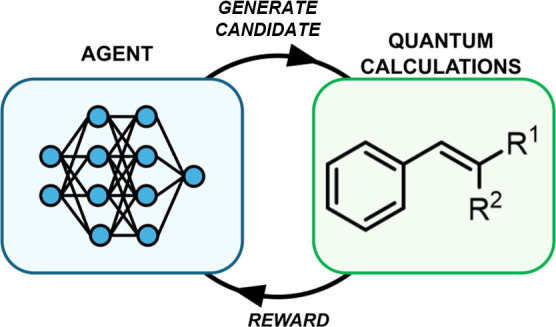

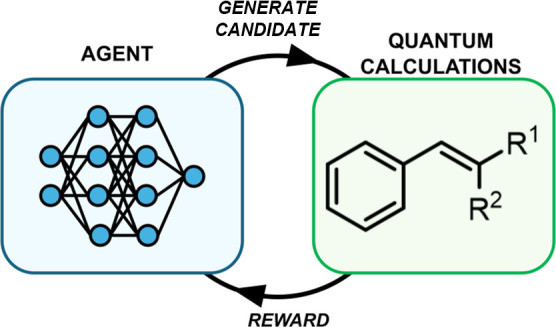

## Introduction

The inverse design (ID) of new molecules is one of the major challenges in chemistry in this century, aiming to generate *de novo* compounds with desired properties.^1–4^ This is a fundamental paradigm shift in computational chemistry that promises a fast discovery of, e.g., new catalysts, drugs, molecular energy storage, and carbon-capturing systems.

However, the complex structure–property relationship in molecules^5^ and the lack of a unified theory to solve this problem limit its development. In fact, the characterization of the chemical space (CS) has to deal with its immense size, which makes its thorough exploration computationally impossible.^6^ Significant efforts in developing evolutionary- and physics-based methods have been reported,^7–13^ while machine learning (ML) methods have recently emerged as powerful tools to accelerate the generation of molecules with predefined properties.^14–20^ Genetic algorithms are among the first approaches to solve inverse molecular design. They are fast statistical methods that mimic Darwin’s evolutionary scheme and do not require any training set for generating candidates. However, they suffer from hyperparameters tuning, and the loss function does not provide informative guidance for improving candidate generation.^11–13^ On the contrary, ML models implement physics-informed loss functions, but are usually based on large data sets that can introduce bias in the exploration of CS. Thus, how to obtain a general, unbiased, i.e., data-free, and computationally feasible exploration method of CS to ID molecules remains an open question.^18^ Among different generative models, those based on reinforcement learning (RL)^21^ are extremely promising for this scope.^18–20^ In RL, an artificial agent learns an optimal policy to exploit a task by interacting with its environment through a trial-and-error procedure. In the ID of molecules, an RL agent learns how to generate molecules that maximize the desired properties, hereafter named the *chemical reward* (*r*_*c*_).

Often compared to RL, Bayesian optimization (BO) algorithms aim to maximize an unknown function, such as the structure–property relationship in molecules rather than learning a generative policy. Although BO does not require a pre-existing data set in principle, in molecular design, it typically relies on a continuous molecular representation (e.g., a latent space learned via data-driven models),^22^ inherently limiting the exploration of molecular candidates to regions of the CS represented by the training set.

Remarkable examples of RL-based data-free generations of molecules have been reported, involving reward metrics based on physicochemical properties, such as drug-likeliness^23^ or lipophilicity,^24–26^ which are not grounded in quantum mechanics (QM), first-principles computations. Recently, QM-driven RL generation of molecules was reported for organic electronic molecular design, although it relied on biased language models trained on databases.^27^ An interesting extension of these approaches—while still data-driven—is surrogate-assisted RL, which integrates an active learning scheme to iteratively train a ML surrogate model to predict the target molecular property.^28^ Therefore, a general and unbiased approach involving QM-driven data-free generation of molecules is lacking, representing a major gap in the field.

In this work, we present an RL approach for data-free molecular ID implemented in the software PROTEUS, employing on-the-fly QM calculations of the target property. PROTEUS includes statistical exploration schemes to escape from local minima as in genetic algorithms,^13^ but leveraging an information entropy model and always informing the generation with target properties. We successfully applied our software to design chemical substituents for a molecular scaffold aiming to maximize the energy difference (or *energy gap*) between two geometrical isomers (or *isomerization energy*) as a toy model of a broader class of inverse design problems focused on tailoring energy gaps, such as catalysts engineering. We demonstrated that PROTEUS allows extensive and effective exploration of highly challenging CSs, including some with up to 2,430,845 syntactically valid combinations, paving the way for a new paradigm in the *de novo* generation of molecules.

## Results and Discussion

### The P-SMILES Syntax and the Isomerization Energy Problem

In the present work, the objective is to maximize the isomerization energy of the double C=C bond of a styrene backbone by inversely designing tailored substituents (see the inset in Figure 1a). The structure–property relationship for such a relatively simple molecular system—i.e., the correlation between the structure of styrene derivatives and the corresponding isomerization energy–is not trivial, since the geometrical isomerization involves a double bond that is conjugated with an aromatic ring. Geometrical isomers thus offer an excellent testbed for inverse design strategies aimed at modulating energy gaps associated with chemical transformations. In fact, isomerization energy prediction is closely related to other molecular design problems, such as catalyst optimization, where the goal is minimizing the energy difference between a rate-determining transition state and reactants.

**Figure 1:**
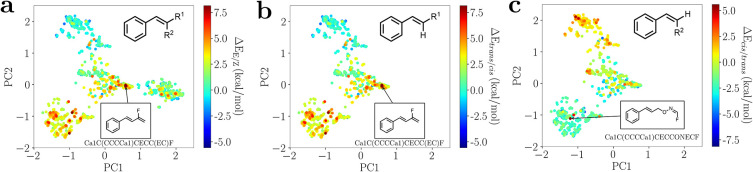
Chemical space analyses and P-SMILES syntax. PCA of (a) the “reference E/Z space”, (b) the *trans*/*cis* subspace (i.e., with *R* ^2^ = H), and (c) the *cis*/*trans* subspace (i.e., with *R* ^1^ = H). The PCAs use Morgan fingerprints of Z molecules with color coding based on the energy gaps computed on DFT-optimized geometries. The structural formula and the P-SMILES strings of the molecules with the largest energy gaps, i.e., Ca1C­(CCCCa1)­CECC­(EC)­F (a and b) and Ca1C­(CCCCa1)­CECCONECF (c), are shown. PCAs were done encoding molecules in bit vectors using the Morgan fingerprint scheme (radius = 5 and 4096 bits), as implemented in the RDKit package.^32^

Molecules are encoded using P-SMILES, an ASCII encoding scheme introduced here to facilitate effective chemical language learning without pretraining based on large molecular databases (see Methods section for details). P-SMILES is a SMILES-based^29–31^ syntax that encompasses a less complex and more compact syntax that mitigates sources of bias during generative RL simulations. P-SMILES simplifies the encoding by limiting to two the maximum number of tokens required to define any structural moiety, i.e., it uses either single- or double-character notation. This reduces significantly the syntactical complexity of encoding geometrical isomers and aromatic rings (Tables S1 and S2 in Supporting Information), removing the sources of bias in the generative RL procedure associated to inequalities involved in the SMILES syntax (see Supporting Information for details). Thus, as detailed in the Methods section, PROTEUS’s generative model is designed to fit the well-defined syntax properties of P-SMILES.

We performed a rigorous assessment of PROTEUS by direct comparison between the set of molecules generated during RL experiments and the corresponding complete space of possible solutions. To keep this comparison computationally feasible, we considered complete chemical subspaces (SubCSs) featuring different dimensions. The largest SubCS fully characterized, hereafter the “reference E/Z space” or simply “E/Z space”, involves all possible sets of *R* ^1^ and *R* ^2^ substituents for the styrene’s backbone resulting from the combinations of maximum 6 P-SMILES tokens. This reference space contains 1628 chemically meaningful pairs of E/Z isomers out of all possible syntactic combinations, i.e., 1,948,716 pairs (see Supporting Information for details). The E/Z isomerization energies of each pair in the “E/Z space” were computed through a multistep routine that involves QM calculations (see Methods section for details).

Interestingly, the distribution of the isomerization energies of the molecular pairs within the space of solutions highlights the complexity of the problem under investigation. Clustering of the molecules with principal component analysis (PCA) shows that the complete set of molecules can be grouped in four (Figure 1a) or three—when *R* ^2^ = H (Figure 1b) or *R* ^1^ = H (Figure 1c)—main clusters with similar molecular features (see Supporting Information for details). In each cluster, however, the distribution of energy gaps is heterogeneous, i.e., clusters contain both positive and negative values.

### Inverse Design of Molecules with PROTEUS

The data-free ID strategy of PROTEUS is depicted in Figure 2. PROTEUS is an RL-based model that generates molecules encoded in the P-SMILES strings. It is based on the proximal policy optimization (PPO)^33^ scheme to learn how to generate new molecules to maximize the outcome of QM calculations. RL solves generative modeling tasks formulated as Markov decision processes, i.e., defined in terms of states, actions, and rewards.^34^ The P-SMILES string encoding a molecule is the state (s_*t*_), and the agent leverages a complex architecture that fits the characteristics of P-SMILES strings. Namely, since a character-based sequential generation could penalize features encoded by two characters, such as cycles and branches, we designed a hierarchical architecture^35^ for molecule generation. As illustrated in Figure 2a, the agent is composed of five neural network (NN) models: (i) a master decides whether to add single-characters or double characters or to end the generation; two positional predictors decide where to place (ii) a single character or (iii) a double character in the current P-SMILES string; and two generators effectively add a (iv) single character or (v) double character. Therefore, the action space is different for each model: the master leverages three possible actions, while both the positional predictors and the token generators return the numerical positions and the vocabulary tokens, respectively, depending on whether they work with single- or double-character. The total reward, *r*_*t*_ (see Methods section for details), recompenses the generated valid molecules considering both the target chemical property (i.e., the isomerization energy), *r*_*c*_, and a chemical diversity index, *r*_*d*_, as follows:

1 $$r_{t}=\alpha r_{c}\left(s_{t}\right)+\beta r_{d}\left(s_{t}\right),$$

where *r*_*d*_ is defined as the reciprocal number of the Tanimoto similarity,^36^ while α and β are hyperparameters (see Methods section for details). Thus, *r*_*c*_ and *r*_*d*_ cooperate in the learning process. In fact, a key ingredient of our generative model is the balance between an efficient exploration of the CS through rewarding the chemical diversity, *r*_*d*_, and proper exploitation of the target chemical reward by maximizing *r*_*c*_.

**Figure 2:**
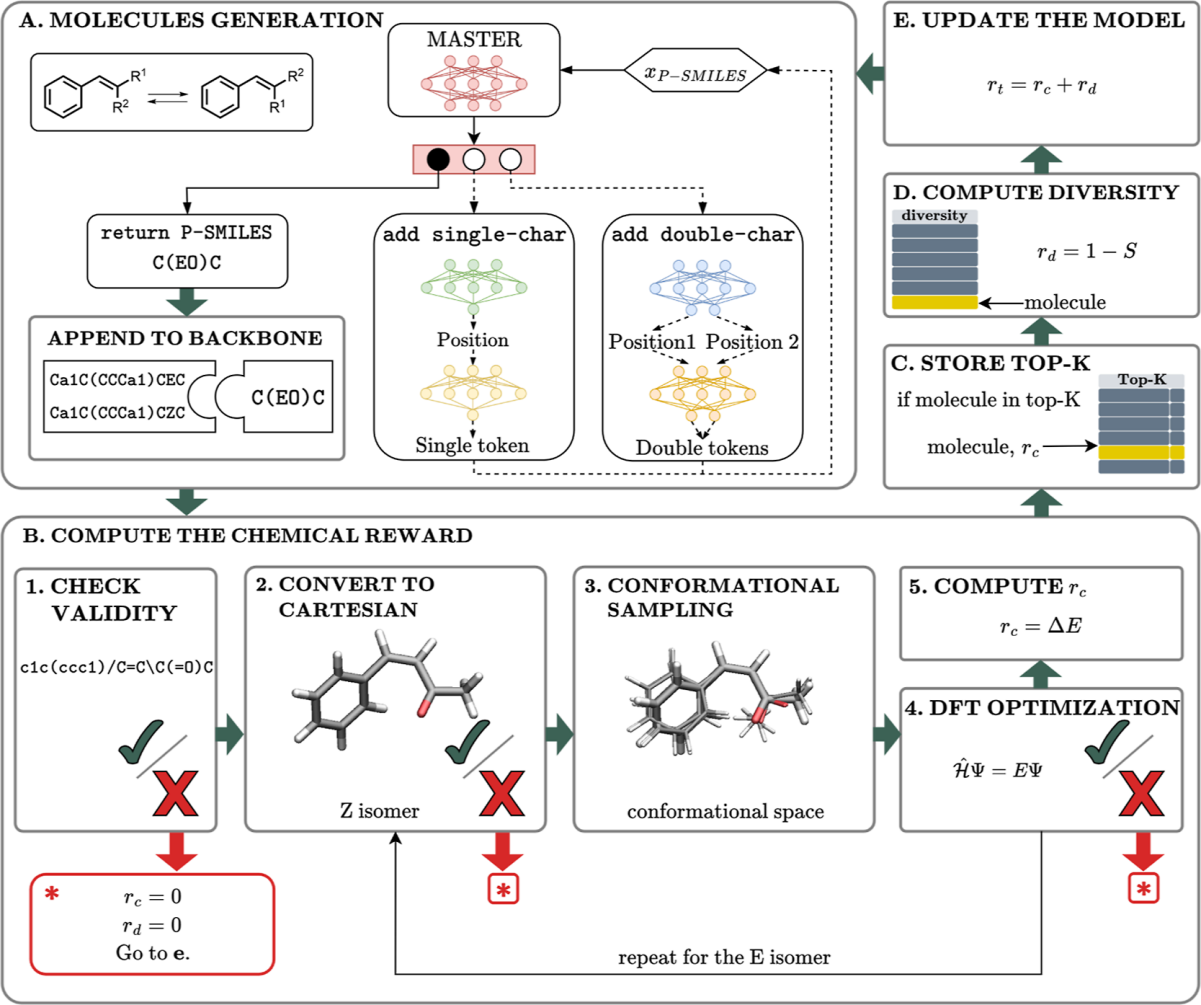
Data-free generation of molecules with PROTEUS. (a) Two substituents, i.e., *R* ^1^ and *R* ^2^ groups, for the styrene backbone (see the inset) are embedded in a P-SMILES string. This string is iteratively generated using a five-model RL agent algorithm, comprising a master decision-maker and single- and double-character predictors. The selected P-SMILES string is then appended to the styrene backbone, as shown for a simple exemplifying case, i.e., *R* ^1^ = COCH_3_ and *R* ^2^ = H for the E isomer (and vice versa for the Z isomer). (b) The molecule’s (i.e., state’s) chemical reward *r*_*c*_ is computed with the following procedure. In step 1, the P-SMILES string is first converted to a SMILES string. Then, if the SMILES has been previously generated, the *r*_*c*_ value is not computed again, moving directly to c; otherwise, a syntactic validity check is performed. If the syntax is not valid, the molecule is considered invalid, and the total reward, *r*_*t*_, is null. If the syntax is correct, the SMILES string is converted to Cartesian coordinates, and it is preoptimized at the MM level. In step 2, a geometry optimization at the DFT-TB level is performed, and if changes in the connectivity occur, the molecule is considered invalid. Otherwise, in step 3, a conformational sampling is performed using metadynamics (MTMD). In step 4, the most stable conformer is optimized at the DFT level. If any structural change occurs, the P-SMILES string is considered invalid. Steps 1–4 are performed for both the E and the Z isomers, and then (in step 5), the E/Z energy gap between isomers (i.e., *r*_*c*_) is computed. (c) If the molecule is among the best K molecules generated so far, it is added (as marked in yellow) to the top-K memory to prioritize training toward more effective solutions. (d) The diversity reward, *r*_*d*_, is computed as the complementary of the Tanimoto similarity (*S*). (e) The total reward is calculated, and the PPO algorithm is used to train the five models.

To push the exploration of unknown regions of the CS, i.e., avoiding the RL generator from being trapped in local minima, an entropy term is added to the loss function (see Methods section for details). The entropy bonus aims to include noise into the generative decision process, and it avoids a deterministic choice of actions, being informed of low-explored regions of the CS. At the same time, the architecture of PROTEUS prioritizes the training toward solutions that have proven to be suboptimal. In fact, PROTEUS stores the top-K P-SMILES strings generated so far, focusing the training on those solutions by doubling their weights in the actual training batch. This type of generator can, thus, focus on both underexplored and high-rewarded regions through the cooperation of various contributions: while the diversity and the entropy terms push the exploration of the CS, the top-K strategy fosters the exploitation of the most promising chemical subspaces.

### Inverse Designing Isomers

#### Inverse Design of E/Z Isomers

Figure 3 shows a representative PROTEUS simulation (simulation 9, Table S5) out of three independent experiments performed for the CS with 6 tokens (simulations 7–9, Table S5) aiming at the maximization of the E/Z energy gap in styrene derivatives obtained by the optimization of the *R* ^1^ and *R* ^2^ substituents (see the inset in Figure 2a), considering *R* ^1^ having always higher chemical priority than *R* ^2^ (for the sake of simplicity). Being asked to walk in a large field of trees while looking for the “best fruits”, thanks to its ML architecture, PROTEUS initially performs a quite broad exploration. In the first 500 epochs, PROTEUS generates molecules featuring *r*_*c*_ values that lie in a broad distribution, i.e., with an energy gap between −4.25 and 7.95 kcal/mol (between −4.23 and 7.96 kcal/mol on average for simulations 7–9), as a direct consequence of random initialization of the policy (Figure 3a). Notably, the quality of this broad exploration is corroborated by an average E/Z energy gap value of *r*_*c*_ (2.85, 2.45, and 3.52 kcal/mol in simulations 9, 8, and 7, respectively; see Table S5) that is close to the average of the whole CS, i.e., 1.33 kcal/mol. The broad exploration is also witnessed by the large value of chemical diversity, *r*_*d*_, of the explored states. In fact, the running average over 10 samples of *r*_*d*_ reaches its maximum value (0.35 for simulation 9; 0.34 on average for 7–9) in these first 500 epochs (Figure 3a). As shown in Figure 3b, the valid P-SMILES strings generated during the first 500 epochs involve molecules belonging to all four clusters of the reference CS.

**Figure 3:**
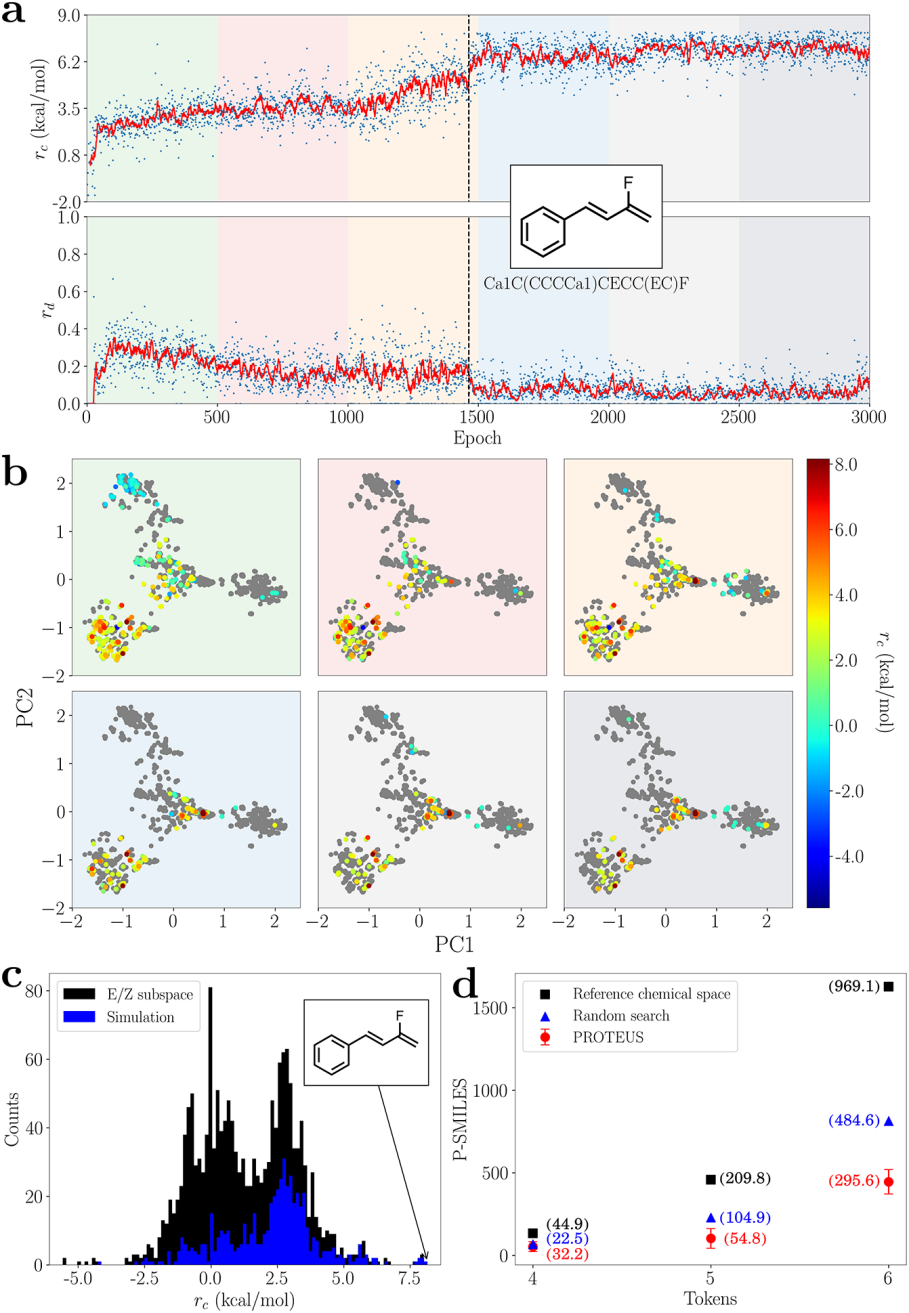
Inverse design of E/Z isomers with PROTEUS. (a) Time-evolution of the chemical and the diversity rewards during a representative PROTEUS simulation for the E/Z isomers within the 6-token CS. Both the mean value of each epoch (blue scatter) and the running average (solid red line) are reported. The epoch corresponding to the first generation of the best solution (with molecular formula and P-SMILES string in the inset), as ranked in the “E/Z space”, is marked with a dashed line. The 3000 epochs reported are divided into 6 windows with different background colors. (b) For each of these 6 simulation windows, the generated molecules are displayed using the principal components defined for the full E/Z space (and reported in Figure 1c). The states generated in each window are labeled using the E/Z energy gap values defined by the color bar, while molecules belonging to the reference CS that are not explored are reported in gray. (c) The E/Z energy gap distributions for isomers in the “E/Z space” (in black) and in the PROTEUS simulations (in blue) are compared. (d) The total number of valid P-SMILES in the CS (black squares) and the average number of valid generations needed to find the best solution using a random search (dark blue triangle) or PROTEUS (red circles) are compared for CS with different sizes (from 4 to 6 P-SMILES tokens). Total average computational time (in hours) required to complete each characterization is reported in parentheses (next to the corresponding markers) keeping the same color scheme. The performance outcomes of PROTEUS reported in panel d are averaged over three independent simulations with three different seeds, and the error bar shows the standard deviation.

In the next 500 epochs, the exploration prioritizes regions featuring larger chemical reward values, with the average *r*_*c*_ value increasing to 3.56 kcal/mol, and reducing the diversity of the states (with *r*_*d*_ averaging to 0.17), as depicted in Figure 3b. In the 1000–1500 epochs region, while keeping a quite constant diversity value in the exploration (with an average *r*_*d*_ of 0.16), PROTEUS largely exploits the chemical reward, with a steep increase in *r*_*c*_ that culminates with the generation of the Ca1C­(CCCCa1)­CECC­(EC)­F state, which is the molecule with the largest E/Z energy gap (i.e., 8.15 kcal/mol) in the full reference space. After finding the very “best fruit” (1500–3000 epochs), PROTEUS mainly exploits the high *r*_*c*_ values with a concomitant drop of the chemical diversity, i.e., it focuses on the best fruits in the best trees. In fact, the average *r*_*c*_ ranges between 6.59 and 7.03 kcal/mol in the 1500–3000 epochs, while the average *r*_*d*_ drops below 0.07 (see Figure 3b). The opposite trend of *r*_*c*_ and *r*_*d*_ can be ascribed to the cooperation between the exploration and exploitation during the learning process. During the exploration phase, when *r*_*c*_ values are low, the impact of *r*_*d*_ on the final value of *r*_*t*_ (eq 1) is not negligible. Instead, when PROTEUS exploits the chemical reward *r*_*c*_, the weight of *r*_*c*_ becomes much larger than *r*_*d*_, thus limiting the impact of the exploration. A similar behavior in the exploitation of *r*_*c*_ was observed in the other simulation replicas but showing different time scales (see Figures S13–S15).

Comparing the distribution of energy gaps in the complete “E/Z space” with those of the states explored by PROTEUS during the 3000 epochs of simulation 9, as depicted in Figure 3c, provides further insights into the learning process. PROTEUS clearly overall prioritizes the generation of valid states with high *r*_*c*_ values, exploring primarily regions with chemical rewards larger than ca. 2 kcal/mol and providing a great computational speed up in the search for the best pair of E/Z isomers. To evaluate the characteristic computational advantage of PROTEUS, we compared it with a random search (without repetitions, i.e., random order) approach for SubCSs composed of 4-, 5-, and 6-token P-SMILES molecules. For each SubCS, three independent simulations were carried out (Table S5). As reported in Figure 3d, to find the best solution, PROTEUS generates a number of unique valid P-SMILES strings that is very close to that of a random search going through all the elements of the set without repetition (i.e., half of the total solutions) only when the size of the CS of valid solutions is small. The 4-token SubCS is composed of 134 unique valid states out of 3770 combinations. With a random order search, 67 valid generations are required on average before sampling the best solution. Similarly, PROTEUS required on average 53 ± 30 unique valid P-SMILES (Table S5). In the cases of the 5- and 6-token spaces, instead, PROTEUS successfully generates the best molecule after generating on average 103 ± 59 and 445 ± 75 unique and valid samples, respectively (Figure 3d). Since the random search approach requires 229 and 814 iterations, respectively, PROTEUS’s results are very satisfactory. In fact, PROTEUS drastically reduces the number of expensive QM property evaluations before finding the best solution and saves on average ca. 70% of computational time for finding the best solution of the 6-token SubCS with respect to a full characterization of the chemical space (Figure 3d and Table S5).

#### Inverse Design of Trans/Cis and Cis/Trans Isomers

The second ID problem we tackled with PROTEUS is the design of a tailored substituent *R* ^1^ that maximizes the *trans*/*cis* energy gap (i.e., the stabilization of the *trans* isomer) for the styrene derivatives, i.e., with the constraint *R* ^2^ = H (Figure 1b). The *trans*/*cis* problem is chemically simpler than the E/Z one, but it could be slightly more complicated from the point of view of the data-free learning process. In fact, the *R* ^2^ = H constraint was set by invalidating those generations that violated it, thus in such a way that it does not affect the total number of combinations of P-SMILES tokens while reducing the density of valid states among them. This reduces the valid pairs of isomers for the 6-token CS from 1628 (in the “E/Z space”) to 1246 (in the hereafter named “*trans*/*cis* space”), making the data-free learning process a bit more challenging. This problem features, by chance, the same best solution as the E/Z one, i.e., Ca1C­(CCCCa1)­CECC­(EC)­F, and the corresponding PROTEUS simulation showed results similar to those of the E/Z simulations, providing additional support for the robust generation performance of PROTEUS (Figure S18). In fact, after a broad exploration of the CS, PROTEUS successfully focuses on maximizing *r*_*c*_ until the generation of the best molecule occurs (Figure S18).

To further assess the capabilities of PROTEUS in balancing exploration and exploitation, we tested the ID routine for the (energetically) reverse ID problem, i.e., the maximization of the *cis*/*trans* energy gap. This task is significantly challenging in the context of the 6-token CS because the best solution has a chemical structure that is similar to molecules with much worse chemical reward, i.e., the “best fruit” is in the “worst tree” (Figure 1c). Despite this, PROTEUS solved the ID problem, confirming the virtuous balance between exploration and exploitation in our implementation (Figure S22). This impressive capability lays the groundwork for future applications of PROTEUS in challenging molecular inverse design tasks.

#### Exploration Beyond a Reference Chemical Space

Given the capabilities of PROTEUS in solving the molecular ID problem, as demonstrated above for fully characterized SubCSs (i.e., with known solutions), we pushed our tool to tackle an ID problem for which the characterization of the full reference space would require a significantly large computational cost (see Supporting Information for details). In particular, we performed the *trans*/*cis* ID simulation by increasing the maximum number of tokens in the generated P-SMILES states from 6 to 7, which raised the total number of possible combinations to 21,435,887, of which 2,430,845 are syntactically valid. To demonstrate effective exploration of such larger CS, PROTEUS should generate (suboptimal) solutions featuring larger (or at least equal) *r*_*c*_ than in Ca1C­(CCCCa1)­CECC­(EC)­F, which is the global solution of the 6-token ID problem. Figure 4 shows the PROTEUS simulation for the 7-token CS. As for the previous simulations, PROTEUS initially performs a broad exploration of the space of solutions, but this exploration period gets longer as the CS increases, as expected. In fact, the average *r*_*d*_ in the first 1000 epochs is constant at ca. 0.25, while the average *r*_*c*_ value is 3.17 kcal/mol. In the subsequent 500 epochs, the average *r*_*c*_ increases to 4.19 kcal/mol, and the Ca1C­(CCCCa1)­CECC­(ECC)­F state is generated. This molecule is quite similar to the best solution of the 6-token CS, differing only for a methyl group, which is actually generated only toward the end of the current 3000 epochs simulation. These two states feature similar *trans*/*cis* energy gaps, whereas the 7-token solution has a slightly higher value, i.e., 8.21 kcal/mol. Remarkably, this shows that PROTEUS can generate highly rewarded 7-token candidates without first solving the 6-token problem and by making almost the same generation effort (i.e., number of epochs <1500) necessary to obtain the best solution of the analogous 6-token ID problem. This demonstrates the great exploration capabilities of PROTEUS. In the next 1500 epochs, PROTEUS focuses on maximizing the *trans*/*cis* energy gap. This is witnessed by an average *r*_*c*_ value of 5.78 kcal/mol and by the fact that the average *r*_*d*_ decreases below 0.16 (see Figures 4 and S21). Within 2500 epochs, the Ca1C­(CCCCa1)­CECC­(EO)­CF state is generated, which is the best solution found in the whole simulation, as it features a *trans*/*cis* energy gap of 9.55 kcal/mol, i.e., 1.40 kcal/mol larger than Ca1C­(CCCCa1)­CECC­(EC)­F. Thus, within 3000 epochs, PROTEUS can find a 7-token solution that is better than the best 6-token solution, demonstrating how effectively PROTEUS can inversely design molecules also in large reference spaces, while targeting chemical reward values computed at the QM level.

**Figure 4:**
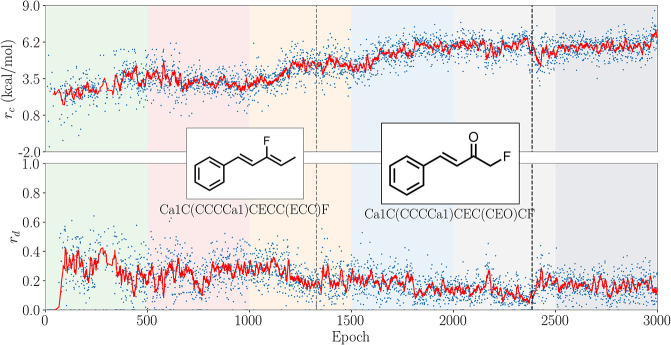
Exploration of a large *trans*/*cis* chemical space with PROTEUS. Time-evolution of the chemical and diversity rewards during a PROTEUS simulation for the *trans*/*cis* isomers within the CS of 7 P-SMILES tokens. Both the mean value of each epoch (blue scatter) and the running average (solid red line) are reported. The epochs corresponding to the generations of the first solution with a *trans*/*cis* energy gap larger than that of the best solution found in the 6-token CS (gray dashed line) and the best solution found along the 3000 epochs simulation (black dashed line) are highlighted, with the corresponding molecular structures and P-SMILES strings in the insets. The 3000 epochs reported are divided into 6 windows with different background colors.

## Conclusions

We proposed an AI tool for data-free *de novo* generation of molecules that involves on-the-fly QM calculations and introduces a tailored ASCII encoding of molecules called P-SMILES. We demonstrated the capabilities of this tool, named PROTEUS, for the molecular ID problem of maximizing the electronic energy gap between geometrical isomers of styrene derivatives. The styrene backbone can isomerize along a double (C=C) bond conjugated with an aromatic ring, resulting in intricate combinations of steric and electronic effects that influence the isomerization energies and add complexity to solving the ID problem. PROTEUS successfully discloses the best solution for a large CS that has been previously fully characterized. The outcome demonstrated that our data-free RL technique applied to molecular ID problems can be successful if a good balance between exploration and exploitation is achieved during the learning process. We stress-tested PROTEUS, indeed, to solve the ID problem for CSs featuring (i) a smaller percentage of valid states with respect to the reference CS or (ii) a best solution with a chemical structure similar to molecules representing the worst solutions. The former problem, associated with the maximization of the *trans*/*cis* energy gap, is a simpler chemical problem than the E/Z one but was constructed in order to feature a less dense space of valid states, leaving PROTEUS with fewer chances to learn the syntactic rules of P-SMILES in the absence of a pretraining. The latter problem is, instead, the reverse energetic problem for the same CS of the former, i.e., the maximization of the *cis*/*trans* energy gap, which requires a virtuous balance between exploration of the space of solutions and exploitation of the task. By solving brilliantly both ID problems, for which the exact solution is known, PROTEUS proved to be robust and to feature enough flexibility to tackle exploration of different CSs, as it can effectively exploit a chemical reward in multiple search directions within a CS.

Considering the computational efforts required by brute-force and high-throughput approaches, PROTEUS provides significant computational savings that allow the exploration of large and complex CSs with first-principles resolution. We further provided evidence for it by tackling the 7-token *trans*/*cis* problem of the styrene derivatives, for which a full characterization of the CS (with up to 2,430,845 syntactically valid combinations) at the QM level would be computationally quite demanding also for computational chemistry laboratories. PROTEUS generated a 7-token (likely suboptimal) solution that features a higher chemical reward than the best solution of the 6 tokens CS while employing a similar number of generations. By progressively identifying better ID solutions at an affordable computational cost, PROTEUS can be readily employed in computational chemistry laboratories. Notably, since the software architecture of PROTEUS can adapt to a specific molecular ID task, it could be applied in the future to exploit other, more complicated ID tasks, opening new avenues to QM-driven ID of molecules in an unbiased, data-free manner.

## Methods

### RL Architecture

The RL model shown in this work consists of five ML models. Each model implements a policy by means of a transformer architecture.^37^ The models are organized as follows: the master, with policy π_*M*_(*s*_*t*_), receives as input the P-SMILES string, *s*_*t*_, produced so far and decides among three actions: (i) add a single-character token to *s*_*t*_, (ii) add double-character token to *s*_*t*_, or (iii) return *s*_*t*_, i.e., ending the generation. If the first action is chosen, *s*_*t*_ is fed into the single-character position predictor, π_*P* ^*S*^_(*s*_*t*_). This predictor outputs a probability vector from which the position for placing a single-character token is sampled. Therefore, the single-character generator, π_*G* ^*S*^_(*s*_*t*_), returns a vector of probabilities from which the single-character token to be placed in the position chosen by the previous NN is sampled, and *s*_*t*+1_ is obtained by modifying *s*_*t*_ accordingly. If π_*M*_(*s*_*t*_) selects the second action, *s*_*t*_ is passed to the double-character position predictor, π_*P* ^*D*^_(*s*_*t*_), which returns two vectors of probabilities, one for each position to be chosen. Thus, the two positions are sampled to ensure always syntactically valid two-character tokens. At this stage, the double-character generator, *π*_*G* ^*D*^_(*s*_*t*_), samples a two-character token, and *s*_*t*+1_ is obtained. Finally, if the last action is selected, then the generation is considered as concluded.

The architecture of PROTEUS overcomes the sequential (i.e., left-to-right) construction strategy of molecules, since it relies on a modification policy similar to the masked language modeling.^38^ In fact, given an intermediate P-SMILES string, i.e., *s*_*t*_, each action can modify *s*_*t*_ by adding a single- or a double-character token in any position. This means, for example, that a chain of carbons CCCCCC could be easily branched (e.g., CCC­(CC)­C) after its construction by a single action, allowing PROTEUS to define complicated structures with single actions.

At the beginning of each PROTEUS simulation, the parameters of each NN are initialized randomly by using the default initializer based on the Glorot uniform distribution. The complete pseudocode for the generative loop is provided in Supporting Algorithms S1 and S2.

Each policy is trained using PPO with prioritized experience replay.^39^ The prioritization scheme implemented in PROTEUS simply doubles the sampling probability for top-K trajectories. The overall loss for each policy is defined as follows:

2 $$L\left(\theta \right)={Ê}_{t}\left[L_{t}^{CLIP}\left(\theta \right)-L_{t}^{VF}\left(\theta \right)+c_{e}S\left[\pi_{\theta }\right]\left(s_{t}\right)\right],$$

with

3 $$L_{t}^{CLIP}\left(\theta \right)={Ê}_{t}\left[\min\left(\frac{\pi_{\theta }\left(a_{t}|s_{t}\right)}{\pi_{\theta_{old}}\left(a_{t}|s_{t}\right)}{Â}_{t},\;clip\left(\frac{\pi_{\theta }\left(a_{t}|s_{t}\right)}{\pi_{\theta_{old}}\left(a_{t}|s_{t}\right)},1-\epsilon ,1+\epsilon \right){Â}_{t}\right)\right]$$

and

4 $$L_{t}^{VF}\left(\theta \right)={Ê}_{t}\left[{\left(V_{\theta }\left(s_{t}\right)-V_{t}^{target}\right)}^{2}\right].$$

*L*_*t*_^CLIP^ is the clipped surrogate objective that modifies the policy toward the maximization of the total reward while preventing too large changes, and *V*_θ_ is the value function used to estimate the value of the current state. *V*_θ_ is computed with another transformer-based NN model identical to the policies described above. The advantage term *A*_*t*_ is estimated using the one-step temporal difference error^40^ as follows:

5 $$A_{t}=r_{t}+\gamma V_{\theta }\left(s_{t+1}\right)-V_{\theta }\left(s_{t}\right),$$

and the value function is trained to approximate

6 $$V_{t}^{target}=A_{t}+V_{\theta }\left(s_{t}\right).$$

*S*[π_θ_] is an entropy bonus that prevents the policy from collapsing over deterministic solution, and *c*_*e*_ is a hyperparameter that weights the entropy term. Eq 7 was used to calculate the entropy of the policy, according to information theory:

7 $$S\left(X\right):=-\sum_{x\in X}p\left(x\right)\log_{b}p\left(x\right)$$

where *X* is our policy vector, *p*(*x*) is the probability of selecting action *x*, and *b* is the number of possible actions. The entropy value *S*(*X*) ranges between 0 and 1. Indeed, when it is maximized, the entropy value of the policy becomes 1, meaning that the policy is completely random, that is

8 $$p\left(x\right)=\frac{1}{b},\text{for},\forall x\in X$$

On the contrary, when *S*(*X*) = 0, the policy is deterministic, and only one action can be selected. In plain words, the entropy is a measure of the exploration capability of our agent in a particular state: the higher the entropy, the higher the exploration. Similarly, according to information theory, high entropy states correspond to low information content value. This underlies the fact that the penalty to pay for a satisfactory exploration of the action space is to lower the confidence of the information content value in the policy.

All the models share the total reward, *r*_*t*_, which is defined as follows:

9 $$r_{t}\left(s_{t}\right)=\{\begin{array}{ll} -1 & \text{if}\;t=T\;\text{and}\;T=0\;\text{or}\;T>L\\ 0 & \text{if}\;t<T\;\text{or}\;s_{t=T\;}is\;\text{not}\;valid\\ \alpha r_{c}\left(s_{t}\right)+\beta r_{d}\left(s_{t}\right) & \text{if}\;t=T\;\text{and}{\;s}_{t\;}is\;valid \end{array}$$

where *t* is a generic step of an episode, *T* is the last one, *L* is the maximum sequence length, *r*_*c*_ is the targeted chemical property, and *r*_*d*_ is a measure of the diversity between the given molecule and *n* molecules generated before, while α and β are hyperparameters that weight each term. Both *r*_*c*_ and *r*_*d*_ are normalized with a discount-based scaling scheme.^41^ A sensitivity analysis of the influence played by the α:β ratio is reported in the Supporting Information (Figures S17–S20, and S23). The complete training algorithm is reported in the Supporting Information.

### Chemical Reward

*r*_*c*_(*s*_*t*_) is a function (or a routine) to compute the desired chemical property of *s*_*t*_, which depends on the chemical structure encoded in the state, *s*_*t*_. This task is exploited by external software for QM calculations. In this work, we focused on the energy gap between geometrical isomers of the same molecule. Once *t* = *T*, the routine to compute *r*_*c*_ is switched on. It comprises different steps to check the validity of *s*_*t*_, based on physical-chemical criteria and QM calculations:

1. The generated P-SMILES string is converted to the corresponding SMILES string. If the conversion fails due to the detection of inconsistency in syntax, *s*_*t*_ is considered invalid.
2. If the SMILES string contains either oxygen–oxygen or nitrogen–nitrogen bonds in linear chain systems, it is considered invalid.
3. The compliance of the basic chemical rules in the SMILES string is verified and, if any rule is broken, the SMILES string is considered invalid. This validity check is done using the RDKit software package.^32^
4. The SMILES string is converted to Cartesian coordinates, and it is optimized at the molecular mechanics (MM) level using the MMFF94 force field (FF),^42^ as implemented in the Pybel module^43^ of the OpenBabel Python library.^44^
5. The molecular total charge is set to zero and, if the molecule is formally not closed-shell, the SMILES string is considered invalid. To check the closed-shell nature of the molecule, we compute the quantity *Q* defined as
10 $$Q=\frac{\sum_{i}n_{i}Z_{i}}{2}.$$
  If *Q* is even, the molecule is considered closed-shell. Otherwise, it is open-shell, with *n* being the number of atoms of element *i* with atomic number *Z*.

6. A second geometry optimization of the structure is done at the DFT-TB level using the xTB software package.^45^ All optimizations have been done using the GFN2-xTB Hamiltonian.^46,47^

7. A geometry check is done on top of the optimized structure to verify that no change in the connectivity occurred during the optimization. The molecule, before and after the optimization, is converted to a graph structure, where atoms are nodes and bonds of any order are single edges. Then, the isomorphism between the graphs is verified. If the two graphs are not isomorphic, the molecule is considered invalid. It is important to highlight that a direct comparison between either SMILES or InchiKey^48^ strings is not a valid choice at this stage since atom typing sometimes changes after the DFT-TB optimization even if the connectivity does not vary. The manipulation of graphs was done using the NetworkX Python library.^49^
8. The conformational analysis is done with the CREST software^50^ on top of the optimized geometry. CREST relies on an automatic MTMD scheme to sample and select conformers of a given molecule.^51^ The simulation time of the MTMD is set three times longer than the default value to improve the exploration of the conformational space. Among the final ensemble of conformers, the conformer with the lowest energy is selected for the next step.
9. The desired molecular property is computed at the selected level of theory. In the present work, we compute the ground-state electronic energy as a single point with DFT-TB or DFT level or after optimizing the geometry of the conformer selected by CREST with DFT. All DFT calculations were carried out with the Gaussian16 software package^52^ and using the exchange–correlation B3LYP functional^53–57^ in pair with the 6-31G­(d,p) basis set for all elements.^58^
10. The InchKey strings of the generated P-SMILES and of the geometry from the previous step are compared to verify that no changes occurred in connectivity, bond orders, or isomerism during the whole routine. Contrary to step 7, working with InchiKey strings is the best choice at this step to ensure an exact correspondence between the generated P-SMILES string, i.e., the state *s*_*t*_, and its total reward value.
11. In the present work, we evaluate the isomerization energy; thus, all the steps are repeated for both E (or *trans*) and Z (or *cis*) isomers. Then, the isomerization energy is computed as the difference between the electronic energies of the two isomers as follows:
11 $$\Delta E_{E/Z}=E_{Z}-E_{E}$$
12 $$\Delta E_{\mathrm{cis}/\mathrm{trans}}=E_{\mathrm{trans}}-E_{\mathrm{cis}}$$
13 $$\Delta E_{\mathrm{trans}/\mathrm{cis}}=E_{\mathrm{cis}}-E_{\mathrm{trans}}$$

### Diversity Reward

The diversity reward, *r*_*d*_(*s*_*t*_), of a given valid molecule is calculated as the reciprocal number of the Tanimoto similarity *S*(·,·)^36^ between the current state and the most similar molecule over the last *n* valid generated molecules,

14 $$r_{d}\left(s_{t}\right)=1-\max_{\forall b\in B_{n}}S\left(a\left(s_{t}\right),b\right)$$

with *n* being a hyperparameter defining the size of the batch *B* of reference molecules previously generated, and

15 $$S\left(a,b\right)=\frac{\left|a\cap b\right|}{\left|a\cup b\right|}.$$

*a* and *b* are the fingerprint arrays to which each molecule (i.e., SMILES string) is embedded, based on given features.^36^ Fingerprints were computed using the MACCS fingerprint as implemented in the Pybel Python library.^43^

### Molecular Encoding with P-SMILES

P-SMILES streamlines SMILES syntax^29–31^ to simplify the syntactic complexity to define isomeric isomers and aliphatic/aromatic rings. Namely, the two- or three-character notations of SMILES of E and Z isomers are substituted with a one-character one, i.e., introducing the E and Z tokens. Similarly, aromatic rings are defined by a specific token an (with n $\in Z^{+}$ ), instead of the conventional representation involving paired numbers and juxtaposed double bonds. P-SMILES, thus, reduces the number of symbols used in SMILES while retaining its encoding capabilities (see SI for details).

### The Reference “E/Z Space”

The “E/Z space” is a complete subspace of valid molecules comprising all the possible E/Z isomers of styrene derivatives, whose substituents are encoded as P-SMILES strings with no more than 6 tokens. The set of tokens we used is the following: [E, Z, a1, 1, #, (), C, N, O, and F].

### Simulations Replica

Three independent E/Z inverse design PROTEUS simulations were carried out for each fully characterized 4-, 5-, and 6-token space (simulation 1–9, Figures S7–S15), using different initial pseudorandom generator seed for neural network weights initialization. This procedure ensured a statistically robust evaluation of the PROTEUS performance.

## Supplementary Material



## Notes

Additional computational details and results, including molecular encoding strategy, implementation details, code architecture, pseudocodes, clustering methods, hyperparameters, QM computations benchmarks, and results of simulations replicas (PDF)
