# Supplementary material for: Quantum Chemistry-Driven Molecular Inverse Design of Stable Isomers with Data-Free Reinforcement Learning
Source: J Chem Theory Comput. 2026 Mar 16;22(7):3373–82. doi: 10.1021/acs.jctc.5c02055 (PMC13085243; doi:10.1021/acs.jctc.5c02055)
Supplement: Supplementary file 1 [file ct5c02055_si_001.pdf]

Supporting Information of Quantum Chemistry-Driven Molecular Inverse Design of Stable Isomers with Data-free Reinforcement Learning

Francesco Calcagno [*,†,‡] Luca Serfilippi [¶] Giorgio Franceschelli [¶] Marco Garavelli [†] Mirco Musolesi [¶,§] and Ivan Rivalta [*,†,‡]

†*Department of Industrial Chemistry, Alma Mater Studiorum University of Bologna, Bologna, 40129, Italy*

‡*Center for Chemical Catalysis - C3, Alma Mater Studiorum University of Bologna, Bologna, 40129, Italy*

¶*Department of Computer Science and Engineering, Alma Mater Studiorum University of Bologna, Bologna, 40136, Italy*

§*Department of Computer Science, University College London, London, WC1E 6BT, United Kingdom*

E-mail: francesco.calcagno@unibo.it; i.rivalta@unibo.it

Contents

S1 Molecular encoding for RL: SMILES *vs* P-SMILES S3

S2 Implementation details S8

S2.1 Neural system architecture S8

S2.2 Pseudocode of PROTEUS S10

S2.3 Computational resources details and parallelization S11

S3 Characterization of the 'E/Z space' S13

S3.1 Benchmark of the QM computations S14

S3.2 Clustering methods S18

S4 Hyperparameters and additional results S23

S4.1 Hyperparameters S23

S4.2 Simulation results summary S24

S4.3 Additional results: the E/Z 4-token simulations S25

S4.4 Additional results: the E/Z 5-token simulations S28

S4.5 Additional results: the E/Z 6-token simulations S31

S4.6 Additional results: the *trans*/*cis* 6-token simulations S34

S4.7 Additional results: the *trans*/*cis* 7-token simulation S37

S4.8 Additional results: the 6-token *cis*/*trans* simulation S38

S4.9 Sensitivity analysis of exploitation and exploration hyperparameters S40

S1 Molecular encoding for RL: SMILES *vs* P-SMILES

One of the most popular encoding for molecule is SMILES, which is based on ASCII strings.[1] More precisely, molecules are defined as sequences of tokens that embed both chemical and structural features. Each token can be compared to a syllabus or a punctuation mark in natural languages since combinations of tokens return informative strings that convey the chemical properties of a system. SMILES is an extremely useful and complete syntax to describe chemical systems,[2–4] which comprises letters, numbers, symbols, and their combinations. SMILES characters could be classified as either *content* or *structural*. Content characters are the symbols of the corresponding chemical element. However, since hydrogen atoms are implicitly considered, the valence of each non-hydrogen atom is always assumed as saturated by H atoms. For example, in the SMILES syntax `C` means methane, while `CC` means ethane. Content characters follow a double syntax and can be written in either capital or small letters. When an element is written in capital letters (e.g. `C`), the valence of its atom is saturated by Hs, i.e. belongs to an aliphatic system (unless followed by other specific characters). Instead, when it is written in small letters (e.g. `c`), it belongs to an (unsaturated) aromatic system. This could turn into the equivalency between `c` and `C=`, or `=C`, where `=` is a structural content for double bonds.

Structural characters can be classified based on the type of structural information encoded. They define, for example, the order of a bond (i.e. `=` if double and `#` if triple), the relative placement of moieties around a double bond (e.g. `X/C=C/Y` to define the E isomer of a `C=C` bond, and `X/C=C\Y` to define the Z isomer of a `C=C` bond), branches, and cycles. Branches and cycles are embedded in pairs of brackets and numbers, respectively. For example, in SMILES syntax tertbutanol is encoded as `CC(C)(C)O`, while methyl clcyclohexane is `CC1CCCCC1`.

Aromatic systems in SMILES naturally feature equivalent definitions, e.g. benzene can be written as either `c1ccccc1` or `C1=CC=CC=C1`. In the context of RL generation where each

action defines the addition of one (or multiple) tokens, in the absence of extensive pretraining, both chemically equivalent strings for benzene are very challenging for generative models since require a long sequence of juxtaposed tokens to be correct. Moreover, in SMILES, aromatic rings are *special cases* in which the modification of just a single character (e.g. `=` in `C1=CC=CC=C1`) can break the aromaticity. This is not the same for the aliphatic rings that usually require more than one string modification to be converted into aromatic ones. Such limitation makes the data-free generation of aromatic rings much more unlikely than aliphatic ones, if SMILES are used.

Similarly, the SMILES generation of an E isomer is (unrealistically) more likely than a Z one. In fact, the E isomer of a double bond can be written using three equivalent notations - i.e. `X=Y`, `/X=Y/`, and `\X=Y\` - while the Z isomer only two, i.e. `/X=Y\` and `\X=Y/`. Notably, SMILES syntax defines `=` as a double bond with *undefined stereochemistry*. However, in practice, when a SMILES string with a double bond with undefined stereochemistry is translated to Cartesian coordinates, it is considered an E isomer by default in several python libraries that implement SMILES.[5,6] All these inequalities in the syntactic complexity of aromatic/aliphatic rings and E/Z double bonds are a possible source of bias in RL-based generative models. Therefore, to leverage the probability of generating those features, we modified the SMILES syntax making it PROTEUS-oriented (P-SMILES syntax). The idea behind P-SMILES is to reduce as much as possible the number of characters, i.e. the possible actions in the model, as well as the number of digits needed to define a syntactic meaning. As reported in Tab. S1, we condensed the definition of E and Z isomers substituting the three-character notation of SMILES with a one-character one, i.e. introducing the `E` and `Z` tokens. Regarding the aromatic rings, we avoided the use of the small-letter notation for chemical elements and introduced a new class of cycles. The original SMILES notation relying on couples of numbers is joined by pairs of numbers marked with an `a`, e.g. `a1`, that define aromatic rings. The new token is specific for aromatic rings and leverages the syntactic complexity of the aliphatic analogs. In fact, if the `Ca1CCCECCa1` P-SMILES is generated, it

is promptly converted to benzene, giving priority to the a1 token rather than the E one.

Overall, PROTEUS reduces the number of symbols used in SMILES, while keeping its encoding capabilities. To summarize, the vocabularies of tokens to define carbon, nitrogen, oxygen, fluorine, single, double, and triple bonds, branches, and rings are:

1. SMILES: [C, N, O, c, n, o, F, /, \, =, #, 1, (,)],
2. P-SMILES: [C, N, O, F, E, Z, #, 1, a1, (,)].

Table S1: **The P-SMILES syntax.** Representative differences between the SMILES and the P-SMILES syntaxes.

Property	SMILES	P-SMILES
E conformation	/X=Y/ or \X=Y\ or X=Y	XEY
Z conformation	/X=Y\ or \X=Y/	XZY
Aromatic ring	C1=CC=CC=C1 or c1ccccc1	Ca1CCCCCa1

Table S2: Comparison between the SMILES and P-SMILES notations to encode geometrical isomers of azobenzene, used here as a general exemplifying case.

Molecules	SMILES	P-SMILES
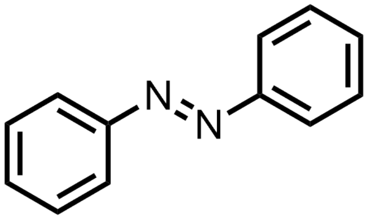	C1=CC=CC=C1/N=N/C1=CC=CC=C1<br>C1=CC=CC=C1\N=N\C1=CC=CC=C1<br>C1=CC=CC=C1N=NC1=CC=CC=C1<br>c1ccccc1/N=N/c1ccccc1<br>c1ccccc1\N=N\c1ccccc1<br>c1ccccc1N=Nc1ccccc1	Ca1CCCCCa1NENCa1CCCCCa1
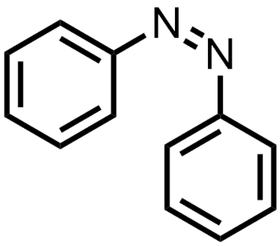	C1=CC=CC=C1/N=N\C1=CC=CC=C1<br>C1=CC=CC=C1\N=N/C1=CC=CC=C1<br>c1ccccc1/N=N\c1ccccc1<br>c1ccccc1\N=N/c1ccccc1	Ca1CCCCCa1NZNCa1CCCCCa1

To verify the performances of the P-SMILES syntax, we carried out a comparative study between P-SMILES and SMILES. For P-SMILES, we imposed that branching characters, i.e. [1, a1, and ( )], are always complete. This means that branching characters appear always

in pairs. Such a constraint mimics the actual generation played by PROTEUS, which is designed to always choose a complete set of those tokens that are meaningful in juxtaposed pairs.

First, we compared the expressiveness of the SMILES and P-SMILES syntaxes. To do this, we benchmarked the ratios between E and Z double bonds and the ratio between aliphatic and aromatic rings in strings randomly sampled for each syntax. All the strings sampled are composed of a random number of tokens comprised between 8 and 20. All the values reported below are averaged over 20 independent batches, each composed of 25,000 samples. The vocabularies used for this test are:

1. [`\`, `/`, `=`, `1`, `C`, `c`] for SMILES,
2. [`E`, `Z`, `a1`, `1`, `C`] for P-SMILES.

As shown in Fig. S1, the SMILES syntax favors double bonds with undefined stereochemistry. Double bonds with undefined stereochemistry are 99.0% of the total, while well-defined E and Z double bonds are 5.7% and 4.3%, respectively. This means that stereochemically labeled double bonds are evenly generated, with a preference for E isomers. When the P-SMILES syntax is turned on, the amount of double bonds with defined stereochemistry increases up to 17.4% for the E isomers and to 17.8% for the Z ones, while the number of double bonds without any specified stereochemistry is 64.8% (Fig. S1A and S1B). The fact that there is a significant number of undefined double bonds is meaningful since all double bonds that are not isomeric (e.g. ketones) cannot have a stereochemical label. The fact that this number decreases from SMILES to P-SMILES suggests that for SMILES, within the set of double bonds with undefined stereochemistry, several are E isomers.

Aromatic rings are rarer than aliphatic ones by definition since a ring is aromatic if it follows the highly specific, Hückel's rule. However, generating an aromatic ring using SMILES is rarer than it should be. As reported in Fig. S1C, within the SMILES syntax the probability that in a random string, a ring is labeled as aromatic is close to zero (0.54%).

Instead, using the P-SMILES syntax, the probability of generating an aromatic ring rises to 8.7% (Fig. S1D).

These outcomes point out the fundamental issue of the bias within the SMILES syntax and, thus, its limitations in RL simulations. Since the isomeric configuration and aromaticity labeling are biased by the use of complicated (and often unequal) combinations of tokens, the exploration of some regions of the space of solution could be limited. While this is particularly relevant for data-free RL generations, it could also be important for generations using a highly representative pretrained chemical language model. Thus, the P-SMILES syntax we developed is more suitable for RL applications since it ideally levels out syntax complexities while limiting the maximum number of tokens needed to define structural isomers and aromatic rings up to two.

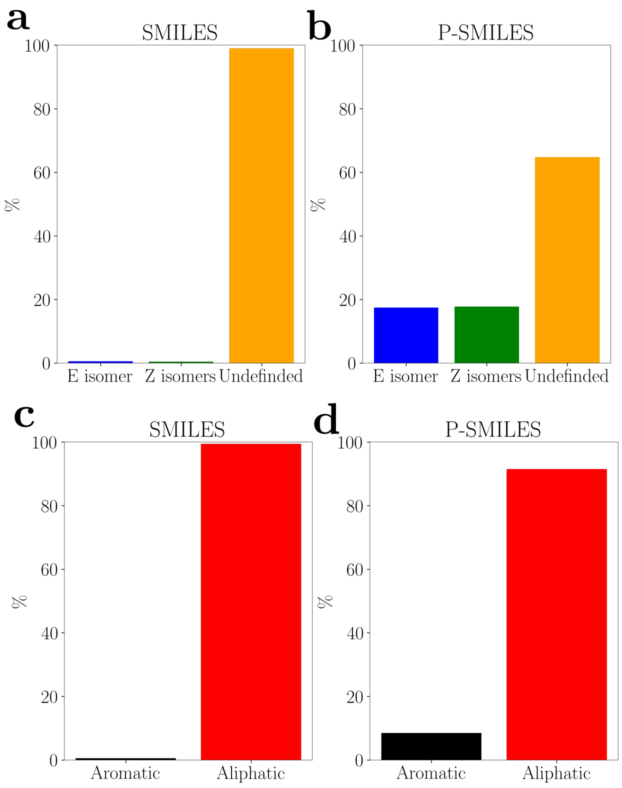

Figure S1: **P-SMILES vs. SMILES.** Relative probabilities of building a double bond with E or Z configuration or an aliphatic or an aromatic ring using (**A** and **C**) SMILES or (**B** and **D**) P-SMILES syntax.

S2 Implementation details

S2.1 Neural system architecture

All neural models in PROTEUS share an initial transformer encoder: given an input sequence, the encoder embeds the sequence of tokens in continuous vectors, adds a positional encoding to characterize the relative position of different tokens, and processes them through a multi-head self-attention layer.[7] The encoder outputs a continuous vector for each token, which is the input information for subsequent agent's models. The master, $\pi_M(s_t)$, receives as input a state s_t and decides among three actions:

1. `add single-character token`
2. `add double-character token`
3. `return` s_t.

If the first action is selected, s_t is passed to the single-character position predictor, $\pi_{P^S}(s_t)$, which returns a vector of probabilities related to all possible positions. In the returned position index i of s_t, a `[MASK]` token is added. Next, the modified s_t is passed to the single-character generator, $\pi_{G^S}(s_t)$, which returns a vector of probabilities related to all the possible single-character tokens. Finally, a token v is sampled and used to replace `[MASK]` in s_t and get s_{t+1}. If the `add double-character` action is selected, s_t is passed to the double-character position predictor, $\pi_{P^D}(s_t)$, which returns two vectors of probabilities, one for each position to be chosen. Two indexes i_0 and i_1 are sampled so that $i_0 :< i_1$. After inserting two `[MASK]` tokens to s_t, each at indexes i_0 and i_1, the double-character generator, $\pi_{G^D}(s_t)$, decides which pair of characters will be inserted by returning a vector of probabilities related to all the possible pairs of characters. The sampled pair v_0, v_1 is then used to replace `[MASK]` tokens and get s_{t+1}. Finally, if the `return` s_t action is selected, the generation is considered concluded. The complete pseudocode for the generative loop is provided in Algorithm 1.

As pictorially depicted in Fig. S2, the master uses a single fully connected layer with softmax activation to return the probability of its three possible actions. The two position predictors share a fully connected layer with softmax activation to return the probabilities of the first position, while only the double-character position predictor uses also a second layer to predict the softmax probabilities of the second position. The single-character generator starts from the encoding of the target token and applies a fully connected layer with softmax activation to get the probabilities of the single characters. Similarly, the double-character generator starts from the encoding of the two target tokens, applies a fully connected layer,

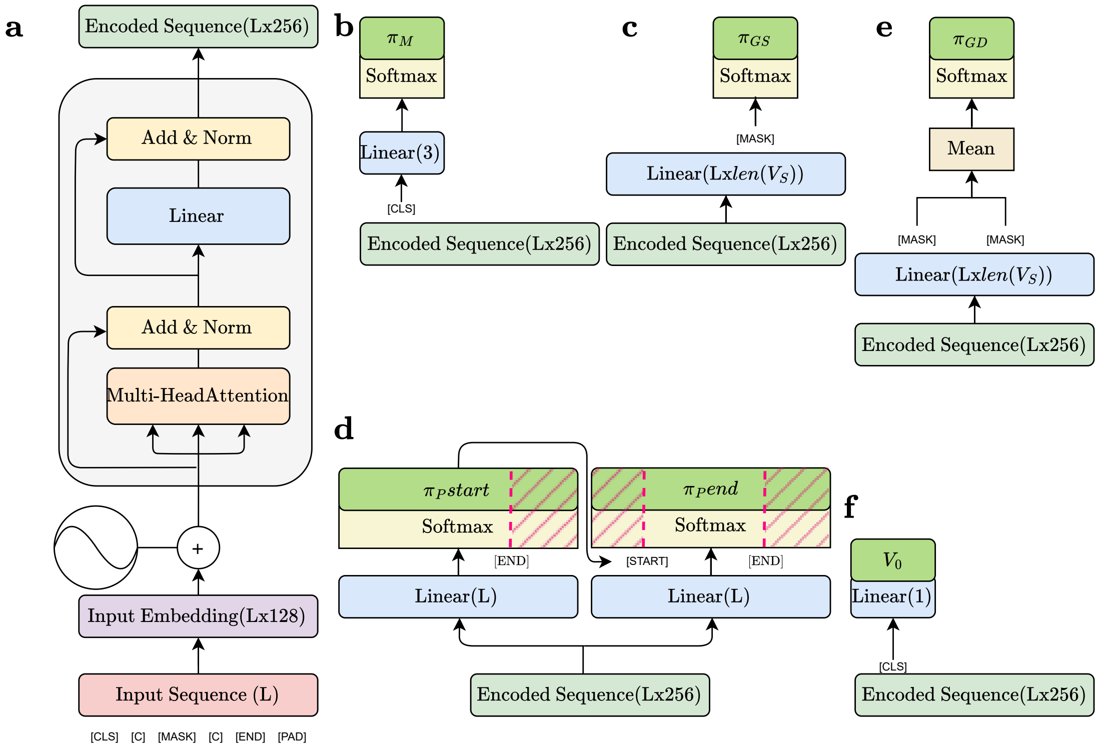

Figure S2: **Neural system architecture of PROTEUS.** The overall architecture of PROTEUS neural system. **a**, The encoder. **b**, The master, M. **c**, The single-character generator, G^S. **d**, The two position predictors, P^D and P^S. **e**, The double-character generator, G^D. **f** , The critic. The input sequence at the token level is marked in red; the encoded sequence as a sequence of continuous vectors is displayed in green; the outputs of the models as probability distributions are reported in lime; the linear layers are highlighted in blue; non-trainable mathematical functions are in yellow; embedding layers are reported in purple, and multi-head attention in orange. The sizes of inputs and NN layers are reported in brackets, with L the length of the generated P-SMILES, i.e. s_t.

and then averages their outputs before applying the softmax activation to return the probabilities for the double characters. Finally, the critic takes the initial encoded sequence and returns the estimate of the value of the current state.

S2.2 Pseudocode of PROTEUS

The pseudocode for generating P-SMILES with our method is reported in Algorithm 1. Instead, the pseudocode for the complete training of PROTEUS models is available in Algorithm 2.

Algorithm 1 P-SMILES generation with PROTEUS

Require: π_M, π_{P^D}, π_{G^D}, π_{P^S}, π_{G^S}, max length L

initialize $s =$ empty_vector
repeat
    $a \sim \pi_M(s)$
    if a is `add double-character token` **then**
        $i_0, i_1 \sim \pi_{P^D}(s)$
        insert `[MASK]` tokens to s_{i_0} and s_{i_1}
        $v_0, v_1 \sim \pi_{G^D}(s)$
        $s_{i_0}, s_{i_1} = v_0, v_1$
    else if a is `add single-character token` **then**
        $i \sim \pi_{P^S}(s)$
        insert `[MASK]` token to s_i
        $v \sim \pi_{G^S}(s)$
        $s_i = v$
    end if
until a is of termination **or** $|s| \geq L$
return s

Algorithm 2 The training procedure of PROTEUS

Require: Backbone b, batch size B, memory size n, max sequence length L, scaling factors α and β

initialize π_M, π_{P^D}, π_{G^D}, π_{P^S}, π_{G^S}, D, top-K
while stop condition not satisfied **do**
  Generate B P-SMILES (S) via Algorithm 1
  Append b to s $\forall s \in S$
  Check validity of s $\forall s \in S$
  for all s **do**
    if s is valid **then**
      Compute fitness r_c of s
      append (s, r_c) to top-K
      sort top-K based on f
      pop last element from top-K
      Compute diversity r_d of s
      if $s \in D$ **then**
        pop s from D
        append s to D
      else if $s \notin D$ **then**
        pop last from D if $|D| = n$
        append s to D
      end if
      $r_t = \alpha r_c(s_t) + \beta r_d(s_t)$
    else if $|s| = 0$ or $|s| > L$ **then**
      $r_t = -1$
    else
      $r_t = 0$
    end if
  end for
  Backpropagate to models prioritizing top-K molecules using the proximal policy optimization (PPO) scheme.[8]
end while
return top-K

S2.3 Computational resources details and parallelization

The PROTEUS simulations follow a complex routine with two main computational bottlenecks: *i*) the training of the NNs, which requires high-performing GPUs, and *ii*) calculations to compute r_c, which are performed on CPUs. All calculations were performed using a hardware setup with 96 CPUs (1 thread each) Intel(R) Xeon(R) Gold 6252N CPU @2.30GHz

and 2 GPUs NVIDIA Tesla T4 (CUDA Version: 12.1, Driver Version: 530.30.02).

To reduce the impact of the cost of QM calculations, the routine to compute r_c is parallelized to run on multithread systems. First, syntactically-valid states are filtered at a low computational cost executing steps 1-3 (Methods section) for all samples generated in a given batch. Thus, PROTEUS generates a variable number of valid P-SMILES strings each epoch, for which the QM energies are computed. In particular, according to the number of threads allocated for the simulation, PROTEUS automatically assigns the maximum number of threads to each isomer and executes steps from 4 to 10 (Methods section) in parallel to compute the QM energies for each candidate molecule. Once all routines running in parallel are completed, PROTEUS computes the energy differences (step 11 in Methods section in the main text), building an array containing the r_c value of each state within the given epoch. In this way, the maximum computational parallelization to compute r_c is always ensured.

S3 Characterization of the 'E/Z space'

The number of possible P-SMILES string combinations for the [`E`, `Z`, `a1`, `1`, `#`, `(`, `)`, `C`, `N`, `O`, `F`] vocabulary comprising up to 6 tokens is 1,948,716, which means 1,948,716 pairs of isomers. 273,718 are syntactically complete and valid pairs. Within this subset, 2,156 pairs of isomers are neutral and closed-shell molecules. Therefore, the number of r_c computations is 4,312. In the most-accurate energy evaluation routine, i.e. when the most stable conformer sampled by CREST is optimized at DFT level, 1,056 did not pass all the validity tests:

1. 4 strings did not be converted to the corresponding Cartesian coordinates, due to OpenBabel inconsistency, i.e. `Ca1C(CCCCa1)/C=C/c1#cn1`, `and` `Ca1C(CCCCa1)/C=C\c1#cn1`, `Ca1C(CCCCa1)/C=C/c1#nc1`, and `Ca1C(CCCCa1)/C=C\c1#nc1`.

2. 119 molecules experienced errors or changes in the number of bonds during the first xTB optimization.

3. 21 molecules were not fully optimized by Gaussian16, mainly due to convergence issues.

4. 912 molecules experienced other kinds of changes in the connectivity at the end of the calculations.

Therefore, the 'E/Z space' finally contains a total of 3,256 styrene derivatives (i.e. 1,628 pairs of E/Z isomers), for which the E/Z energy gap was computed. The *trans*/*cis* (or *cis*/*trans*) subspace of the 'E/Z space', i.e. with R^2=H, features 1,246 out of these 1,628 pairs, while containing 1,644 pairs of syntactically valid strings associated with neutral and closed-shell molecules. Notably, when the maximum length of the generated P-SMILES is increased by just one token, i.e. from 6 to 7, the number of syntactically valid closed-shell pairs of *trans*/*cis* isomers that should be validated at the QM level increases, from 1,644 to 6,005. Given the computational cost of the QM-based validity routine, comprising a rigorous conformational sampling, as evaluated for the 4-, 5-, and 6-token CSs (see Fig. S3) and the

additional cost that will be introduced by including an extra token, i.e. involving larger and more flexible molecules, the computational effort to fully characterize the 7-token CS with 12,010 *trans*/*cis* molecules becomes significantly large.

S3.1 Benchmark of the QM computations

Since the chemical reward adopted in this work is an isomerization energy computed at the QM level, benchmarking this estimated quantity is mandatory. In this section, thus, we report a basic benchmark of the QM methods used in this work to compute the chemical rewards. However, it is crucial to note that targeting high accuracy in the computation of the isomerization energies is not within the scope of this work. In fact, here, we aimed to develop an unbiased RL tool able to broadly explore large CSs and properly exploit the chemical property associated with the involved molecular states, independently from the specific values of their chemical rewards and, thus, from the level of the QM method used. PROTEUS generation capabilities are conditioned by its ML architecture, while the quality of the molecules generated, i.e. their potential use in practical applications, depends on the level of theory chosen for computing r_c. Thus, in the following, benchmark studies are reported to set the ground for future practical applications of PROTEUS.

The final electronic energy of the most stable conformer (according to CREST simulations) of the molecules generated by PROTEUS was computed at three different levels: *i*) DFT-TB optimized geometries, *ii*) DFT single-point calculations on top of *i*), or *iii*) DFT optimized geometries. The isomeric energies estimated by these methods were compared (Fig. S3), using the energies of DFT-optimized geometries as reference.

First, we compared the DFT-TB and single-point DFT energies. As shown in Fig. S3b, the correlation is poor. Moreover, the energies computed using DFT-TB range between -1 and 3 kcal/mol ca., while the DFT energies are distributed in a much larger range, i.e. between -5 and 8 kcal/mol. Further analysis gave insights into the difference between DFT energies computed for geometries optimized with DFT-TB and DFT. Namely, we investi-

gated both the quality of DFT-TB geometries and, thus, their relative single-point DFT energies. As reported in Fig. S3a, the energies are almost linearly correlated (R^2=0.80). The correlation is better when the maximum root mean square deviation (RMSD) value between the geometry of DFT- and DFT-TB-optimized geometries is little, i.e. ca. $<$0.50 Å. In contrast, molecules that exhibit large energy differences before and after DFT optimization, generally feature large RMSD values ($>$ 1.25 Å). For few geometries, single-point calculations overestimate the energy gap by more than 15 kcal/mol (Fig. S3a). Even if rare, such a behavior is potentially very dangerous for the learning process, since it would lead to over-rewarding some molecules and introduce bias.

To assess the overall computational cost of QM calculations, which are the actual computational bottleneck in PROTEUS, the average time required for CREST simulations and DFT optimization was analyzed. Fig. S3c shows that the largest effort is required for the indispensable conformational analysis. CREST calculations require more than double the time of DFT optimizations. In the case of the 6-token subset of molecules, the computational cost to sample the conformers is ca. 5 times higher than the DFT optimization (Fig. S3c). Not surprisingly, the total computational cost increases as a function of the length of P-SMILES strings, since the number of molecular degrees of freedom increases as well, making the conformational space to explore wider.

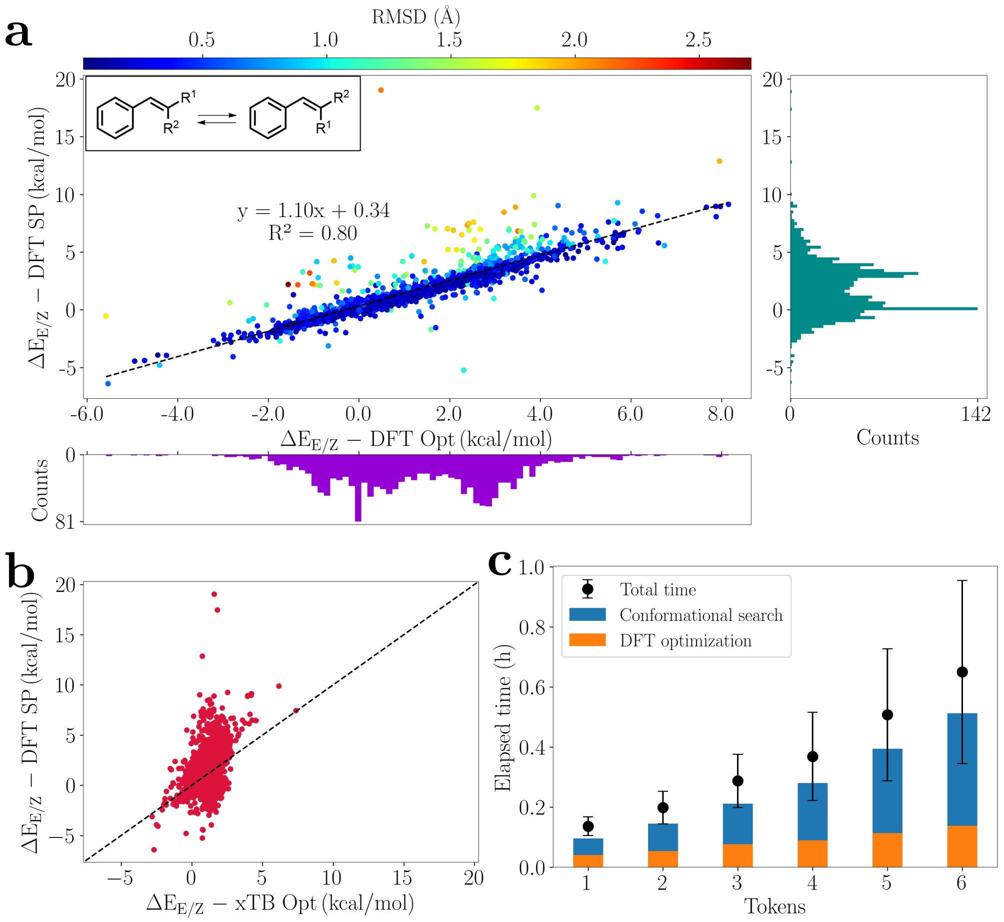

Figure S3: **E/Z energy gap correlations and computational costs.** **a**, Linear regression analysis of the E/Z energy gaps in the 'reference E/Z space' computed by single-point DFT calculations of DFT-TB optimized geometries and DFT geometry optimizations. Color coding is based on the maximum RMSD between DFT-optimized and non-optimized geometries. The distributions of E/Z energy gaps are reported for both single-point calculations (light cyan histogram, right panel) and DFT-optimized geometries (dark violet histogram, bottom panel). **b**, Correlation between energy gap computed with GFN-xTB and B3LYP/6-31G(d,p) single-point on top of the conformational search for molecules in the 'reference E/Z space'. **c**, Average time for the conformational search calculations with the CREST software (blue) and the DFT optimization with the Gaussian16 software (orange) for different numbers of tokens using 6 CPUs. Results are reported only for valid pairs of isomers in the 'reference E/Z space'.

Notably, the absolute and relative energy values are changing whenever dispersion, entropic, and solvation effects are included or the basis set is varied. In the following, the effect of these corrections is described for the best and worst 5 molecules, as ranked in the reference 'E/Z space'. On top of the geometry optimized with DFT, the final energy was refined with the following protocols: *i*) the thermal correction was computed at room tem-

perature with B3LYP/6-31G(d,p), *ii*) the basis-set correction was included by computing the single-point energy at the B3LYP/6-311++G(2d,2p)[9–12] level of theory, *iii*) the effect of the dispersion was calculated by computing the single-point energy at the B3LYP-D3(BJ)/6-311++G(2d,2p)[13,14] level of theory, iv) the effect of the solvation correction was estimated by computing the single-point energy at the B3LYP-D3(BJ)/6-311++G(2d,2p)/SDM(water)[15] level of theory.

As reported in Tab. S3, the thermodynamics of the best and worst 5 molecules is consistent for all the methods, i.e. no changes in the sign of the energy gaps occur. However, the relative ranking is highly dependent on the computational protocol. This outcome is not surprising, since any change, e.g. the presence of an implicit solvent, may lead to non-negligible modifications in the local electron density resulting in an extra-stabilization of some isomers. For example, when dispersion and solvent corrections are included, the energy gap of `Ca1C(CCCCa1)CECC(EO)C` varies significantly (from 7.97 to 5.60 kcal/mol) likely due to the presence of the electron-withdrawing group C=O, which is sensitive to variations in the environment. Similarly, dispersion and solvation corrections reduce the energy gap.

Even if different computational protocols turn into different rankings of the same set of molecules, it is important to mark again that it does not affect the machinery of PROTEUS nor its capability to generate candidates that successfully maximize a property. Obviously, all the important factors mentioned above should be taken into consideration for practical applications of PROTEUS to ID problems.

Table S3: **Comparison of computational protocols.** Comparison of the effects of different quantum chemistry protocols in estimating the E/Z energy gap. SBS = 6-31G(d,p), BBS = 6-311++G(2d,2p).

P-SMILES (R^1/R^2)	$\Delta E_{E/Z}^{B3LYP/SBS}$	$\Delta G_{E/Z}^{B3LYP/SBS}$	$\Delta E_{E/Z}^{B3LYP/BBS//B3LYP/SBS}$	$\Delta G_{E/Z}^{B3LYP/BBS//B3LYP/SBS}$	$\Delta E_{E/Z}^{B3LYP\text{-}D3(BJ)/BBS//B3LYP/SBS}$	$\Delta G_{E/Z}^{B3LYP\text{-}D3(BJ)/BBS//B3LYP/SBS}$	$\Delta E_{E/Z}^{B3LYP\text{-}D3(BJ)/BBS/SDM(water)//B3LYP/SBS}$	$\Delta G_{E/Z}^{B3LYP\text{-}D3(BJ)/BBS/SDM(water)//B3LYP/SBS}$
**Top 5**								
`C(EC)F`	8.15	8.25	7.82	7.92	6.56	6.66	6.97	7.07
`C(EO)C`	7.97	7.94	7.03	6.99	5.37	5.33	5.64	5.60
`CCENOC`	7.96	10.09	8.72	10.85	6.51	8.64	4.79	6.92
`CCCENO`	7.88	8.85	8.71	9.68	5.43	6.40	3.88	4.85
`C(EC)O`	7.87	8.06	8.03	8.21	6.80	6.99	7.12	7.30
**Worse 5**								
`(NEO)O`	-4.44	-4.10	-4.04	-3.71	-4.12	-3.78	-3.48	-3.15
`OCNOC`	-4.73	-5.23	-5.04	-5.54	-3.83	-4.33	-1.71	-2.21
`OCNOCN`	-4.95	-5.58	-5.63	-6.26	-3.91	-4.54	-1.80	-2.43
`(F)NOF`	-5.54	-4.62	-6.16	-5.24	-5.91	-4.99	-6.97	-6.06
`CONZF`	-5.58	-4.80	-6.73	-5.95	-6.31	-5.53	-4.54	-3.76

S3.2 Clustering methods

The 'visualization' of complex and large CS is a key point for the interpretation of the learning process and of the ID results. Standard procedures require *i*) encoding molecules (i.e. SMILES or P-SMILES strings) as bit vectors according to the Morgan fingerprint scheme,[16] and then *ii*) reducing the space dimension of the dataset to allow visualization and improve interpretation.

In the present work, three different methods to analyze the 'E/Z space' are compared: *i*) the principal component analysis (PCA),[17] *ii*) the T-distributed Stochastic Neighbor Embedding (t-SNE),[18,19] and *iii*) the Uniform Manifold Approximation and Projection (UMAP).[20,21] Each tentative of clustering the elements of the 'E/Z space' reported here was carried out on Z isomers (using Morgan fingerprints with radius = 5 and 4,096-bit).

PCA is a widely used methodology to reduce the data dimensionality. The performance of PCA in reducing the number of dimensions is evaluated by computing the fraction of the overall variance recovered by the m principal components chosen for the reduction. Ideally, this fraction should be close to 1, since it estimates how much of the original information is preserved after the transformation. However, in the case of data with high dimensionality, like in the case of 4,096-bit Morgan fingerprints, the fraction of the overall variance recovered by the first two components is low, i.e. ca. 10% in the present case. Therefore, PCA could

appear to be not adequate for the analysis of the ‘E/Z space’, at least at first instance. As already discussed in the main text, the clustering of the ‘E/Z space’ with PCA leads to four main clusters, which become three when the space is filtered by excluding those molecules with R^2=H (Figg. 1a and 1b in the main text). This outcome clearly shows that a 2-component PCA actually already distinguishes a key structural characteristic of the molecules in the CS, as the chemical species of R^2. Similarly, by plotting the diversity value between a random reference molecule and the remaining ones, it is clear that chemically similar molecules belong to the same cluster, while molecules that are chemically different usually belong to different clusters (Fig. S4). As discussed in the main text, given the complex structure-property relationship investigated, the correlation between the clusters and the energy gap, in our specific case, is already gratifying.

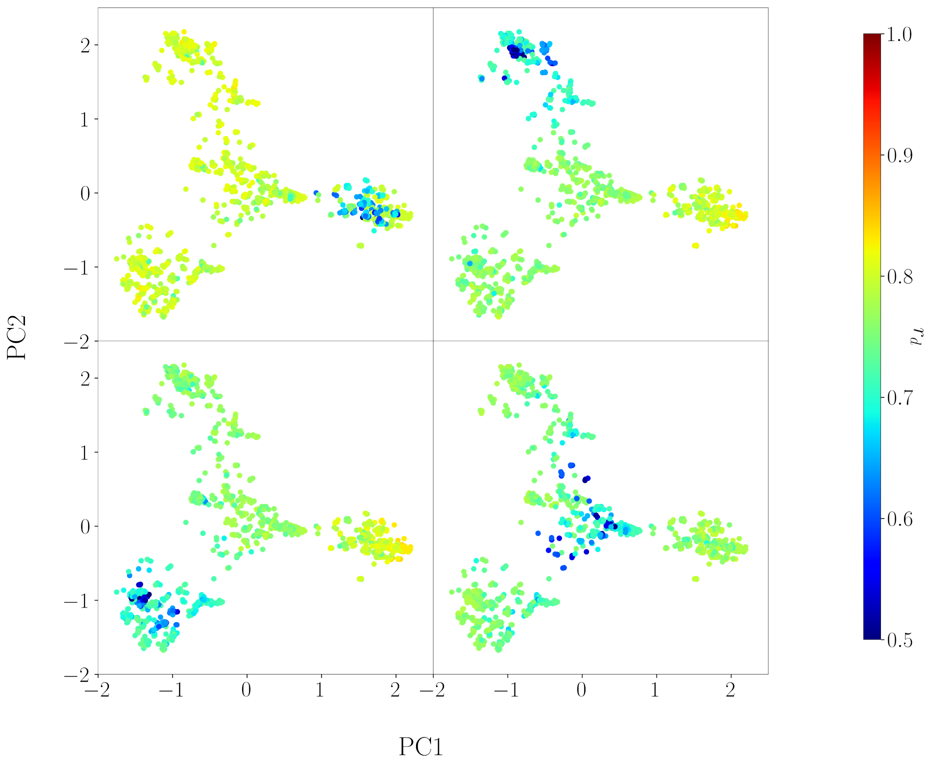

Figure S4: **PCA clusters and diversity value correlation.** PCA clustering of the ‘E/Z space’ based on Morgan fingerprint of four Z molecules (one for each panel). The color coding in each panel is associated with the diversity values of all molecules in the ‘E/Z space’ computed using a random molecule as reference. Molecules with a diversity value below 0.5 are labeled with a diversity value equal to 0.5.

In fact, the quality of the relationship between the clusters and the property does not depend only on the performance of the clustering method, but mainly on the capability of the chosen encoding, i.e. the Morgan fingerprint, to correlate to the property itself. In plain words, the correlation is lacking if the encoding method does not contain any relevant information to describe the property.

Given the limited variance covered by the PCA with 2 components, results were compared with the t-SNE and the UMAP methods. Since t-SNE is a highly computationally demanding method, it is often used in conjunction with PCA. In fact, to save computational time, the number of dimensions of the original dataset is often reduced with a m-components PCA choosing m such that a significant part of the overall variance is recovered. Then, on top of PCA-transformed data, the t-SNE reduction is performed to improve the visualization of the l-dimensions data, preserving the local structure of data. Fig. S5a shows the dimension reduction carried out with t-SNE on top of a PCA analysis done by choosing enough components to recover 95% of the overall variance. The figure shows that t-SNE leads to more and better-shaped clusters than PCA. However, similarly to previous analysis, the relationship between the clusters and the energy gap is not perfect and some clusters show both large and small energy gaps. Interestingly, even if 95% of the overall variance is recovered, similar results are obtained as for the reference PCA. In fact, by coloring the t-SNE clusters in dark red or blue according to the conditions $R^2 \neq H$ or $R^2 = H$, respectively, a clear separation between those two sets is highlighted, as observed for PCA (Fig. S5b). To summarize, the t-SNE reduction is less compact than PCA, without offering any further information.

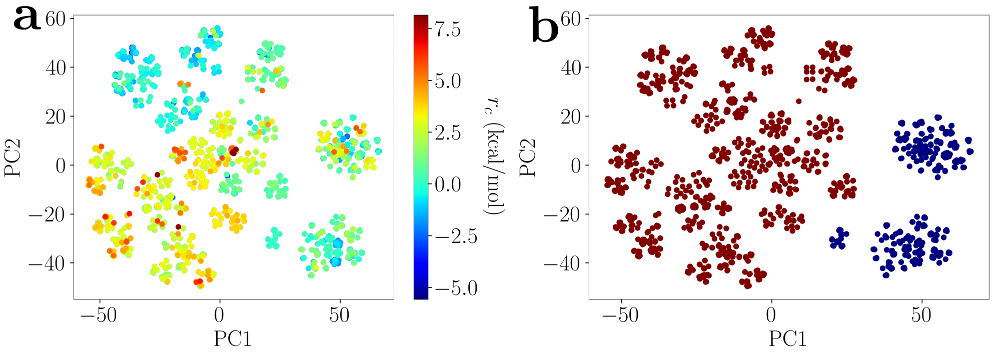

Figure S5: **t-SNE analysis.** Clustering of the 'reference E/Z space' based on Morgan fingerprint of Z molecules and using the t-SNE algorithm. The clusters are colored according to (**a**) the energy gaps computed on DFT optimized geometries, and (**b**) the chemical structure of the R^2 moiety, i.e. dark red if $R_2 \neq$H and blue is R^2=H.

Similarly to t-SNE, UMAP is a promising dimensionality reduction technique for datasets with high-dimension data. Since the outcome of UMAP is highly affected by the choice of hyperparameters, the optimization of the two principal hyperparameters, i.e. the number of neighbors and the minimum distance between data points, was carried out. As shown in Fig. S6, the shape of clusters dramatically changes according to the choice of different hyperparameters. Moreover, in none of the dimension reductions reported in Fig. S6 a clear improvement in the description of the structure-property relationship is reached with respect to t-SNE or PCA.

Thus, PCA provided a robust and fast transformation of the 'E/Z space' data. t-SNE is a good clustering method, but it does not retrieve any additional information with respect to PCA for this case, while increasing the computational cost of the analysis. For the analysis of the 'E/Z space', the UMAP did not improve either the interpretation of the actual distribution of molecules in different clusters, and the choice of hyperparameters is far from trivial.

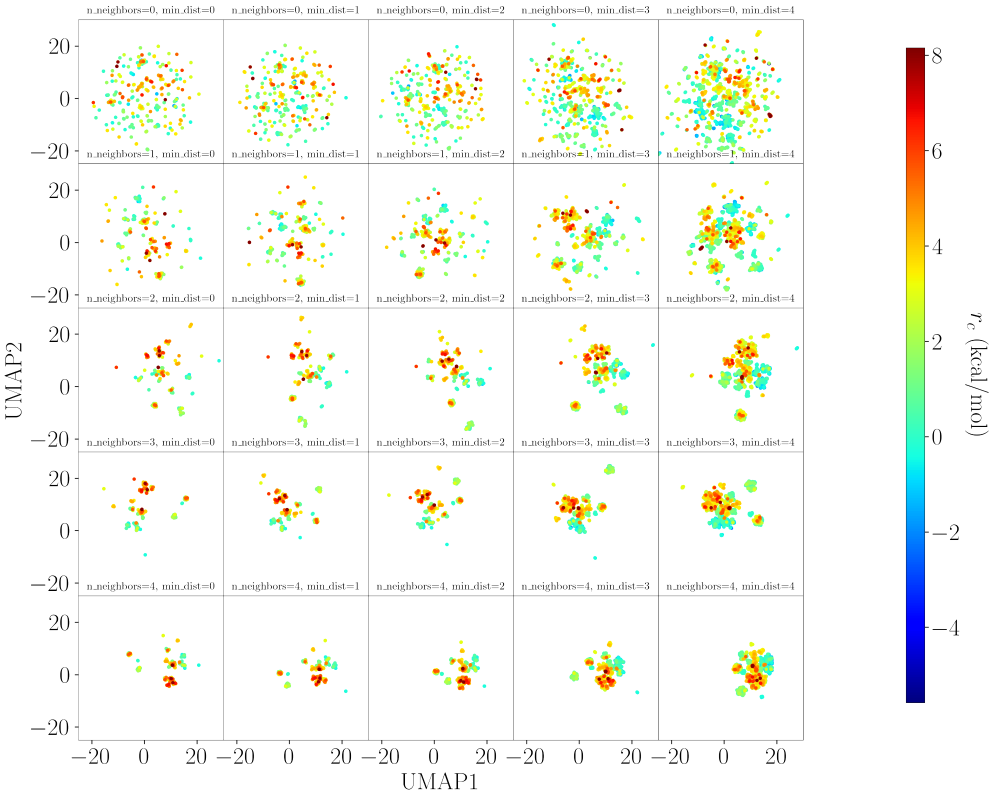

Figure S6: **UMAP analysis.** Screening of the dependence on the number of neighbors and the minimum distance hyperparameters of the UMAP algorithm in clustering the 'reference E/Z space' based on Morgan fingerprint of Z molecules. The clusters are colored according to the energy gaps computed on DFT-optimized geometries.

S4 Hyperparameters and additional results

In the following, details regarding the hyperparameters used in each PROTEUS simulation and additional results on the time evolution of the density of valid solutions and of the entropy of each model are reported.

S4.1 Hyperparameters

Tab. S4 reports the hyperparameters used for all the simulations discussed in the present work.

Table S4: Full list of hyperparameters used in each PROTEUS experiment.

Name	Symbol	Value					
Backbone-E	-	`Ca1C(CCCCa1)CEC`					
Backbone-Z	-	`Ca1C(CCCCa1)CZC`					
Vocabulary single	V_S	[`Z`, `E`, `#`, `C`, `F`, `N`, `O`]					
Vocabulary double	V_D	[`()`, `11`, `a1a1`]					
Tokens	L	4	5	6	6	6	7
Seed	-	[1, 2, 3]	[1, 2, 3]	[1, 2, 5]	1	1	1
Batch size	B	16	16	16	16	16	16
Learning rate	η	1.e-5	1.e-5	1.e-5	1.e-5	1.e-5	1.e-5
Discount	γ	1	1	1	1	1	1
PPO clip coefficient	ϵ	0.2	0.2	0.2	0.2	0.2	0.2
Master entropy coefficient	c_{eM}	5.e-3	5.e-3	5.e-3	5.e-3	6.e-3	6.e-3
Position models entropy coefficient	c_{eP}	1.e-3	1.e-3	1.e-3	1.e-3	1.e-3	1.e-3
Single generator entropy coefficient	c_{eG^S}	4.e-2	4.e-2	4.e-2	4.e-2	4.e-2	4.e-2
Double generator entropy coefficient	c_{eG^D}	1.e-2	1.e-2	1.e-2	1.e-2	1.e-2	1.e-2
SMILES memory length	n	15	15	15	15	15	15
Top SMILES memory length	K	3	3	3	3	3	3
Coefficient r_c	α	1	1	1	[0, 1, 2]	1	1
Energy gap	ΔE	E/Z	E/Z	E/Z	*trans*/*cis*	*cis*/*trans*	*trans*/*cis*
Simulation ID	-	[1, 2, 3]	[4, 5, 6]	[7, 8, 9]	[13, 10, 14]	11	12

S4.2 Simulation results summary

Tab. S5 summarizes results on Simulations 1-9. Since PROTEUS implements an adaptive parallelization scheme that maximizes the number of CPUs used for computing reward each generation batch (Sec. S2.3), the CPU time saving strongly depends on each simulation and sampling progression. For this reason, we opted to report average epoch time assuming the mean CPU time required for quantum chemistry calculations as we recorded during the characterization of the full EZ reference space plus the average update time of the model. Interestingly, the computational cost for model update is negligible on single epochs with respect to quantum chemistry calculations. In fact, model update requires ca. 0.01-0.02 hours, while quantum chemistry calculations' time demand ranges between 0.34 to 0.60 hours for 4- and 6-token spaces, respectively.

Table S5: **PROTEUS simulations.** Results of independent RL simulations replicas. The number of epochs played before generating the best state as ranked in the 'E/Z space', the number of unique states (u) generated before generating the best solution of the CS considered, and its average over the replicas ($\bar{u} \pm \sigma$), with σ the standard deviation, are reported for the 4-, 5-, and 6-token E/Z PROTEUS simulations. Average time per epoch is reported for each simulation.

Simulation	Number of tokens	First epoch best	Unique valid state (u)	$\bar{u} \pm \sigma$	Average epoch time (hours)
1	4	1872	96		
2	4	2920	27	53±30	0.35
3	4	631	36		
4	5	304	18		
5	5	401	145	103±59	0.47
6	5	1332	145		
7	6	5055	533		
8	6	544	352	445±74	0.61
9	6	1464	451		

S4.3 Additional results: the E/Z 4-token simulations

Additional results on the E/Z 4-token simulations are reported below.

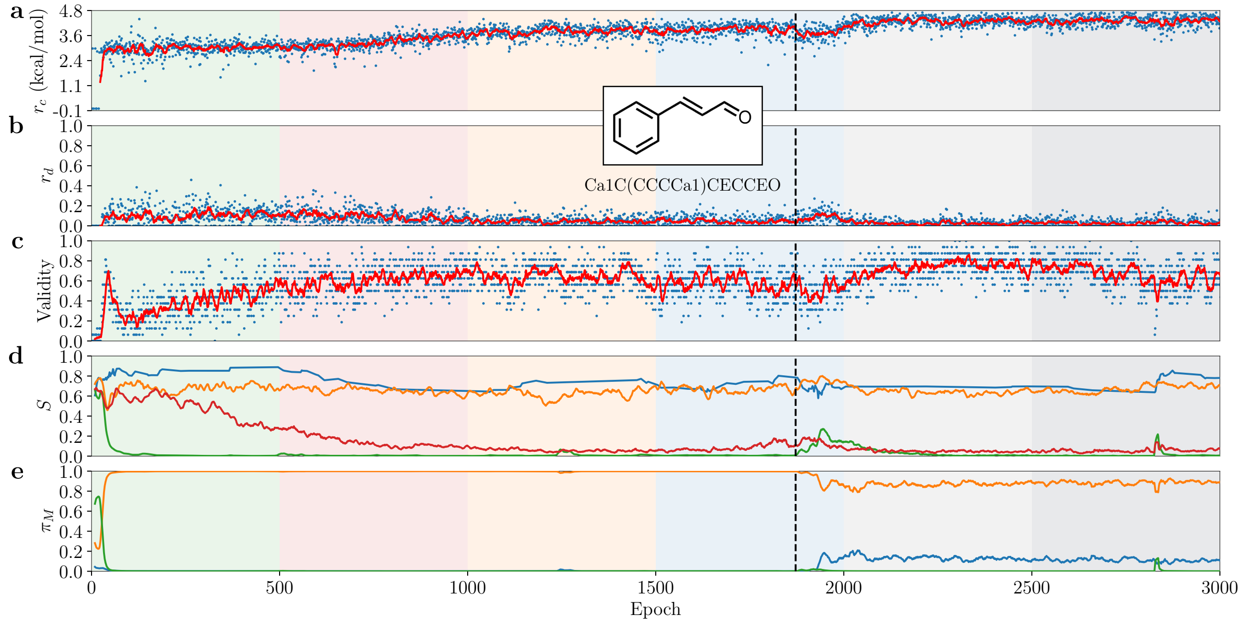

Figure S7: **Simulation 1.** Time-evolution of (**a**) the chemical and (**b**) the diversity reward, and (**c**) the validity density. Both the mean value of each epoch (blue scatter) and the running average (solid red line) are reported. Time-evolving average of the (**d**) entropy value of each policy of the models - M (blue), G^S (yellow), G^D (green), P (red) - and the (**e**) policy value of the master M, `add double-char` (blue), `add single-char` (yellow), `return state` (green). The epoch corresponding to the first generation of the best molecule (inset), as ranked in the 'E/Z space', is marked with a dashed line. The total epochs shown in each panel are divided into 6 subsets, as highlighted by different color backgrounds.

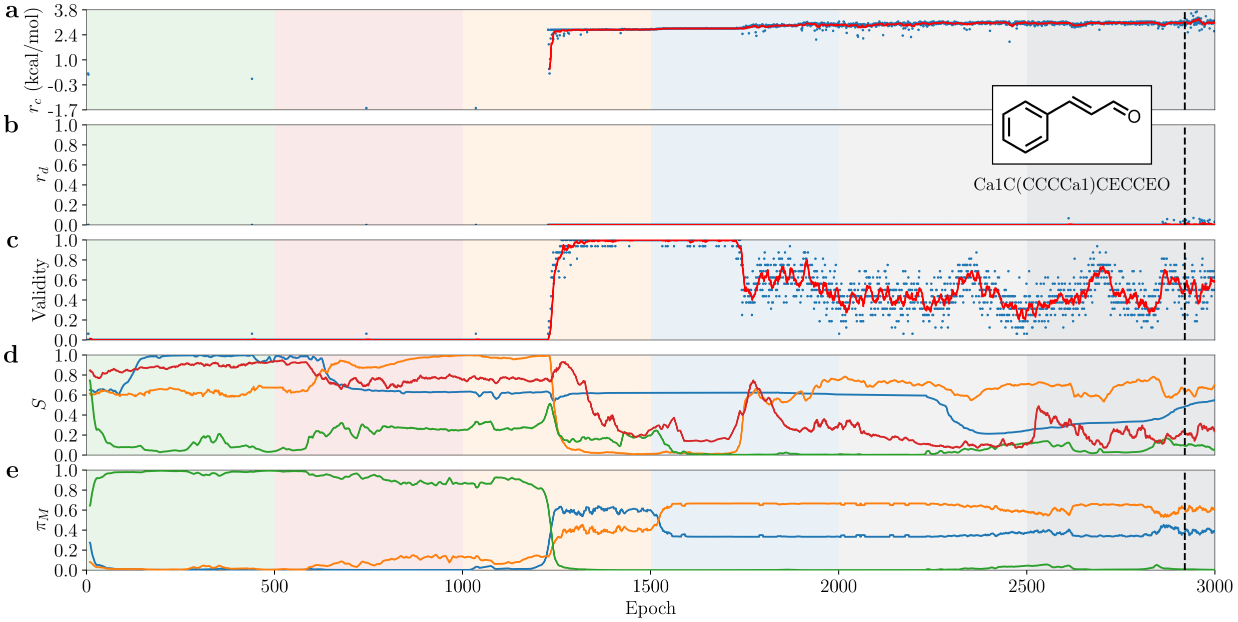

Figure S8: **Simulation 2.** Time-evolution of (**a**) the chemical and (**b**) the diversity reward, and (**c**) the validity density. Both the mean value of each epoch (blue scatter) and the running average (solid red line) are reported. Time-evolving average of the (**d**) entropy value of each policy of the models - M (blue), G^S (yellow), G^D (green), P (red) - and the (**e**) policy value of the master M, `add double-char` (blue), `add single-char` (yellow), `return state` (green). The epoch corresponding to the first generation of the best molecule (inset), as ranked in the 'E/Z space', is marked with a dashed line. The total epochs shown in each panel are divided into 6 subsets, as highlighted by different color backgrounds.

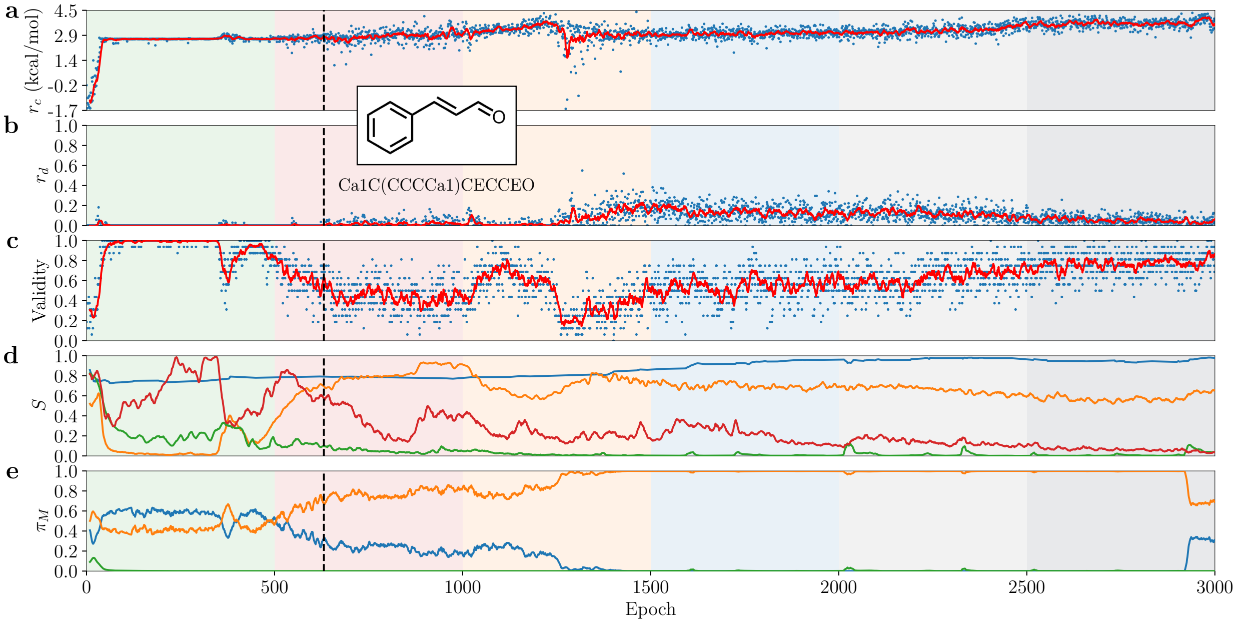

Figure S9: **Simulation 3.** Time-evolution of (**a**) the chemical and (**b**) the diversity reward, and (**c**) the validity density. Both the mean value of each epoch (blue scatter) and the running average (solid red line) are reported. Time-evolving average of the (**d**) entropy value of each policy of the models - M (blue), G^S (yellow), G^D (green), P (red) - and the (**e**) policy value of the master M, `add double-char` (blue), `add single-char` (yellow), `return state` (green). The epoch corresponding to the first generation of the best molecule (inset), as ranked in the 'E/Z space', is marked with a dashed line. The total epochs shown in each panel are divided into 6 subsets, as highlighted by different color backgrounds.

S4.4 Additional results: the E/Z 5-token simulations

Additional results on the E/Z 5-token simulations are reported below.

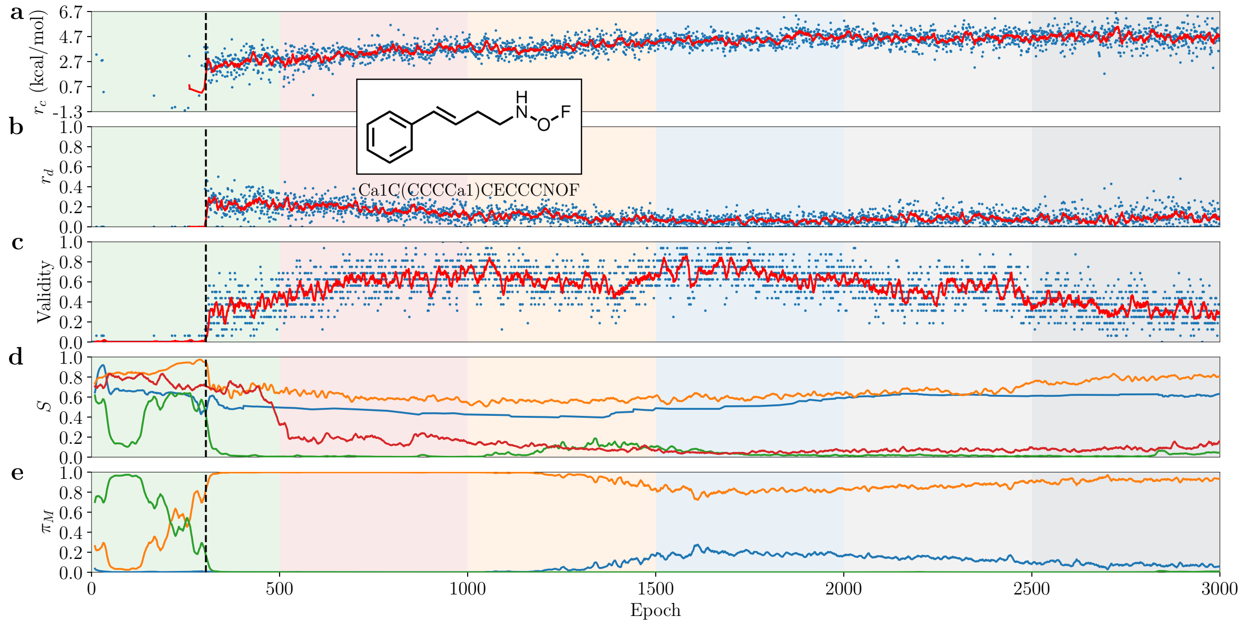

Figure S10: **Simulation 4.** Time-evolution of (**a**) the chemical and (**b**) the diversity reward, and (**c**) the validity density. Both the mean value of each epoch (blue scatter) and the running average (solid red line) are reported. Time-evolving average of the (**d**) entropy value of each policy of the models - M (blue), G^S (yellow), G^D (green), P (red) - and the (**e**) policy value of the master M, `add double-char` (blue), `add single-char` (yellow), `return state` (green). The epoch corresponding to the first generation of the best molecule (inset), as ranked in the 'E/Z space', is marked with a dashed line. The total epochs shown in each panel are divided into 6 subsets, as highlighted by different color backgrounds.

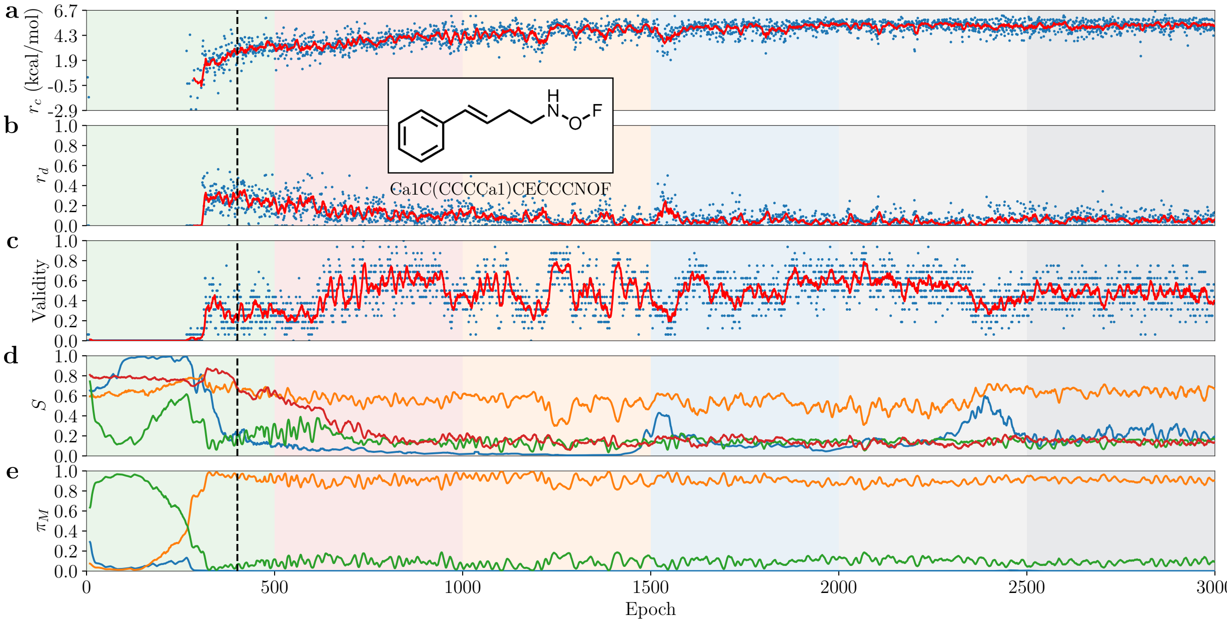

Figure S11: **Simulation 5.** Time-evolution of (**a**) the chemical and (**b**) the diversity reward, and (**c**) the validity density. Both the mean value of each epoch (blue scatter) and the running average (solid red line) are reported. Time-evolving average of the (**d**) entropy value of each policy of the models - M (blue), G^S (yellow), G^D (green), P (red) - and the (**e**) policy value of the master M, `add double-char` (blue), `add single-char` (yellow), `return state` (green). The epoch corresponding to the first generation of the best molecule (inset), as ranked in the 'E/Z space', is marked with a dashed line. The total epochs shown in each panel are divided into 6 subsets, as highlighted by different color backgrounds.

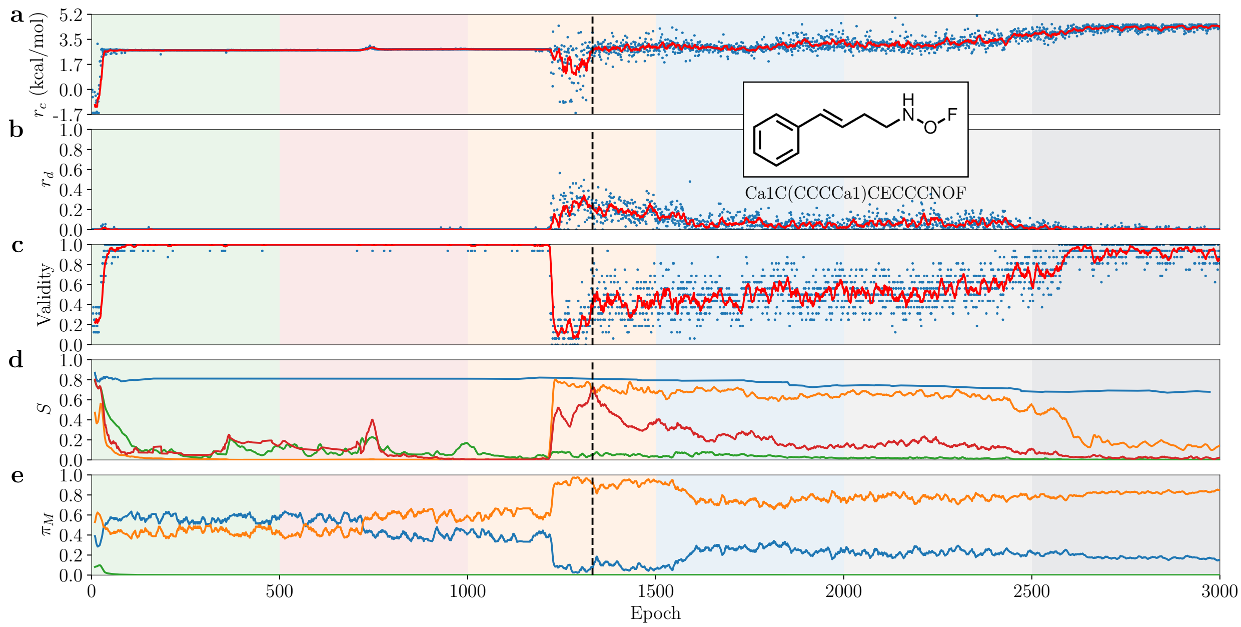

Figure S12: **Simulation 6.** Time-evolution of (**a**) the chemical and (**b**) the diversity reward, and (**c**) the validity density. Both the mean value of each epoch (blue scatter) and the running average (solid red line) are reported. Time-evolving average of the (**d**) entropy value of each policy of the models - M (blue), G^S (yellow), G^D (green), P (red) - and the (**e**) policy value of the master M, **`add double-char`** (blue), **`add single-char`** (yellow), **`return state`** (green). The epoch corresponding to the first generation of the best molecule (inset), as ranked in the 'E/Z space', is marked with a dashed line. The total epochs shown in each panel are divided into 6 subsets, as highlighted by different color backgrounds.

S4.5 Additional results: the E/Z 6-token simulations

Additional results on the E/Z 6-token simulations are reported below.

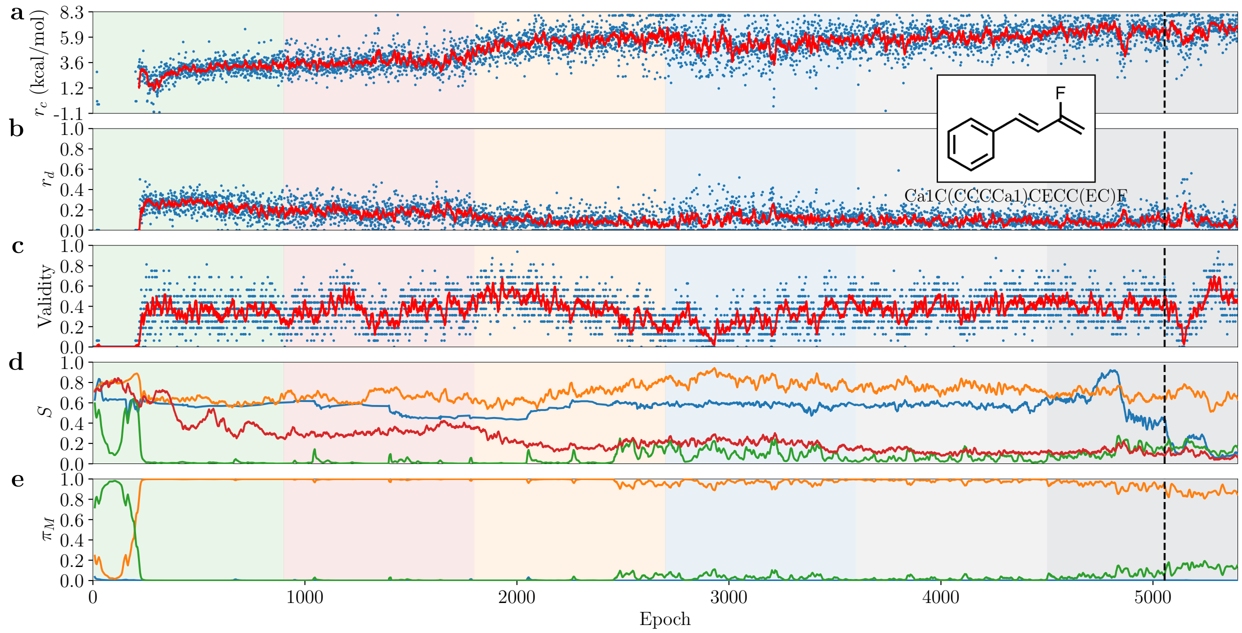

Figure S13: **Simulation 7.** Time-evolution of (**a**) the chemical and (**b**) the diversity reward, and (**c**) the validity density. Both the mean value of each epoch (blue scatter) and the running average (solid red line) are reported. Time-evolving average of the (**d**) entropy value of each policy of the models - M (blue), G^S (yellow), G^D (green), P (red) - and the (**e**) policy value of the master M, `add double-char` (blue), `add single-char` (yellow), `return state` (green). The epoch corresponding to the first generation of the best molecule (inset), as ranked in the 'E/Z space', is marked with a dashed line. The total epochs shown in each panel are divided into 6 subsets, as highlighted by different color backgrounds.

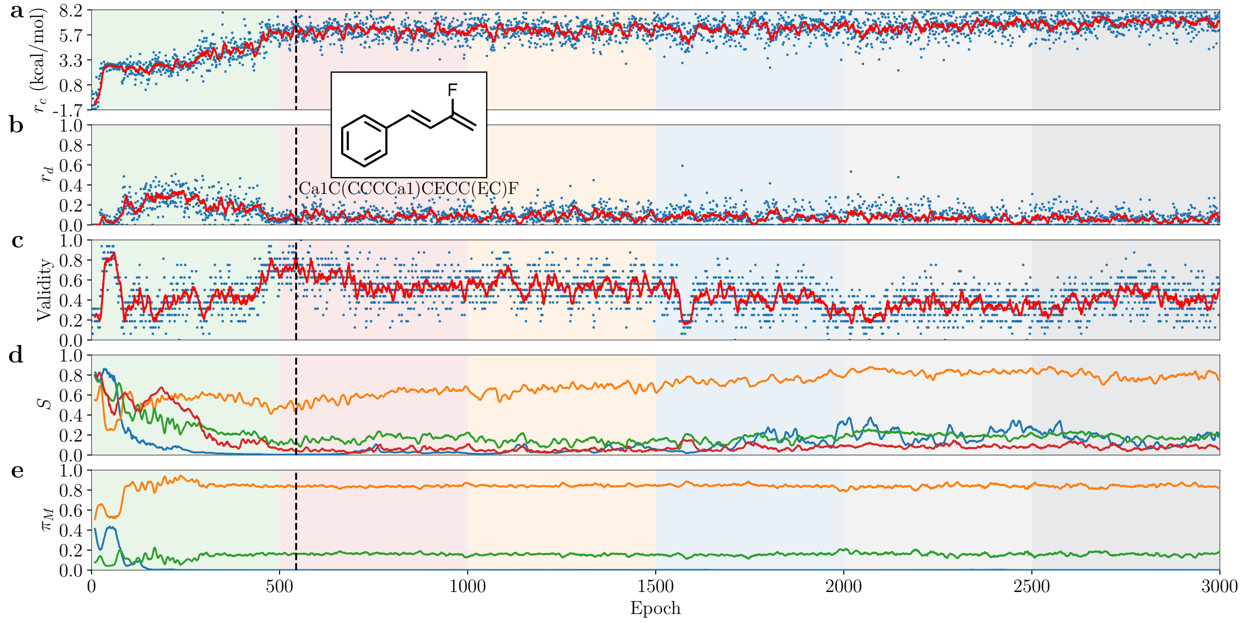

Figure S14: **Simulation 8.** Time-evolution of (**a**) the chemical and (**b**) the diversity reward, and (**c**) the validity density. Both the mean value of each epoch (blue scatter) and the running average (solid red line) are reported. Time-evolving average of the (**d**) entropy value of each policy of the models - M (blue), G^S (yellow), G^D (green), P (red) - and the (**e**) policy value of the master M, `add double-char` (blue), `add single-char` (yellow), `return state` (green). The epoch corresponding to the first generation of the best molecule (inset), as ranked in the 'E/Z space', is marked with a dashed line. The total epochs shown in each panel are divided into 6 subsets, as highlighted by different color backgrounds.

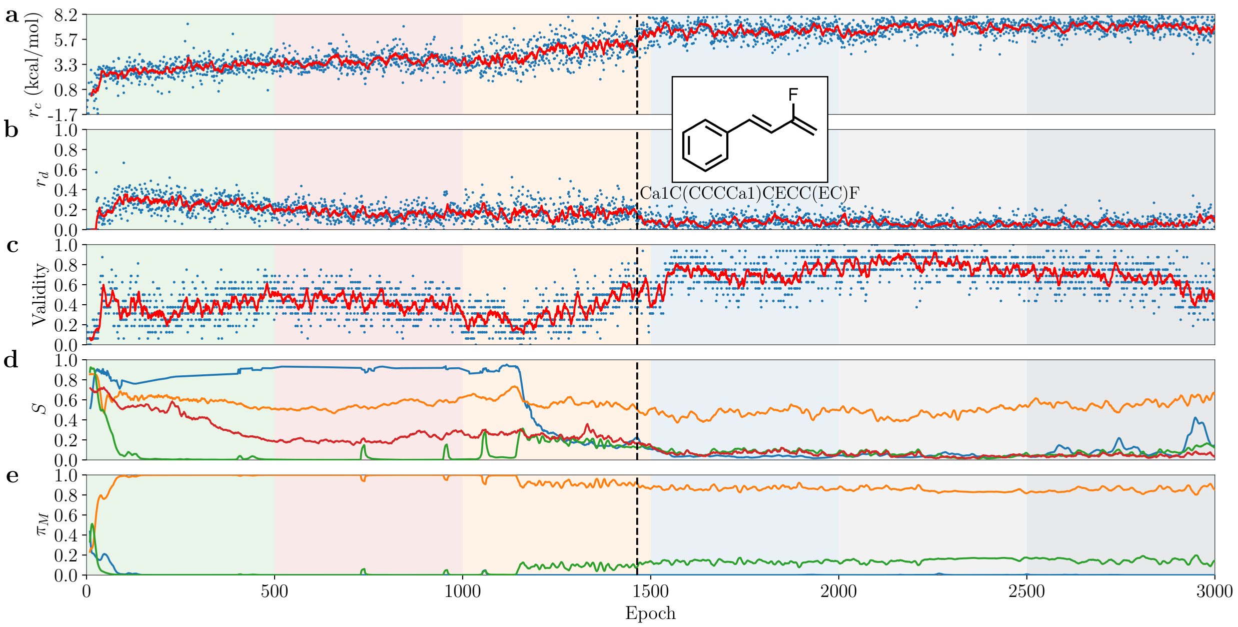

Figure S15: **Simulation 9.** Time-evolution of (**a**) the chemical and (**b**) the diversity reward, and (**c**) the validity density. Both the mean value of each epoch (blue scatter) and the running average (solid red line) are reported. Time-evolving average of the (**d**) entropy value of each policy of the models - M (blue), G^S (yellow), G^D (green), P (red) - and the (**e**) policy value of the master M, `add double-char` (blue), `add single-char` (yellow), `return state` (green). The epoch corresponding to the first generation of the best molecule (inset), as ranked in the 'E/Z space', is marked with a dashed line. The total epochs shown in each panel are divided into 6 subsets, as highlighted by different color backgrounds.

S4.6 Additional results: the *trans*/*cis* 6-token simulations

Additional results on the *trans*/*cis* 6-token simulations are reported below.

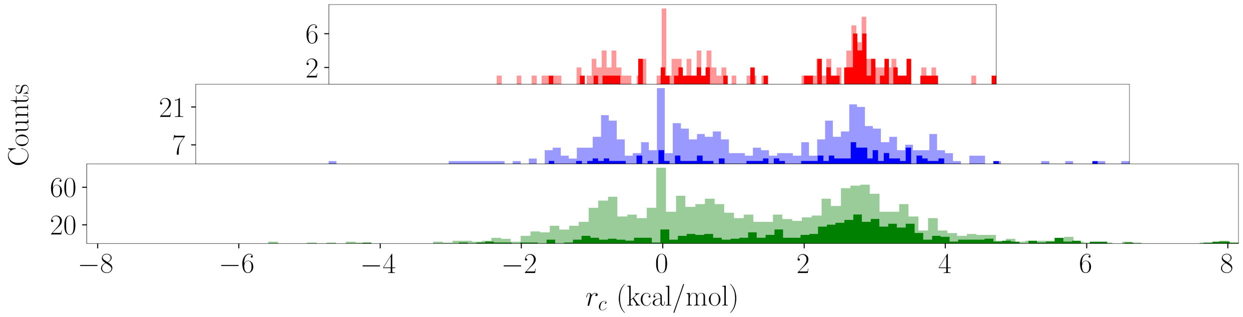

Figure S16: **Explored sub-spaces**. Explored chemical space (solid) VS reference chemical space (shadowed) for 4 (red), 5 (blue), and 6 (green) token sizes of the P-SMILES strings.

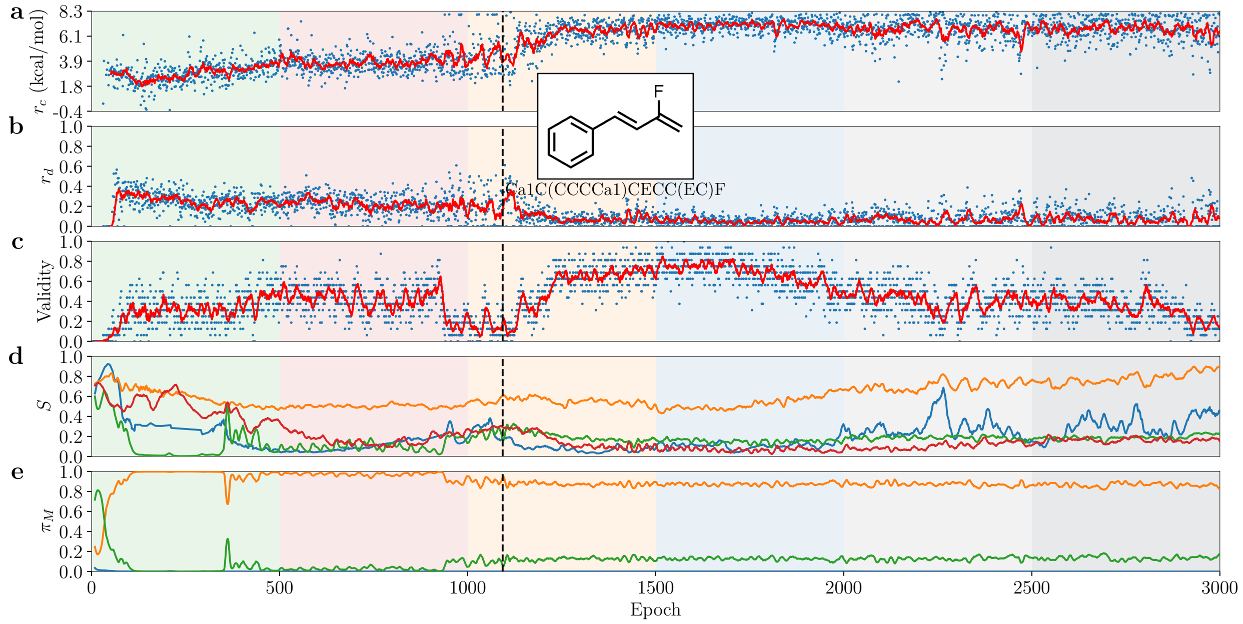

Figure S17: **Simulation 10.** Time-evolution of (**a**) the chemical and (**b**) the diversity reward, and (**c**) the validity density. Both the mean value of each epoch (blue scatter) and the running average (solid red line) are reported. Time-evolving average of the (**d**) entropy value of each policy of the models - M (blue), G^S (yellow), G^D (green), P (red) - and the (**e**) policy value of the master M, `add double-char` (blue), `add single-char` (yellow), `return state` (green). The epoch corresponding to the first generation of the best molecule (inset), as ranked in the 'E/Z space', is marked with a dashed line. The total epochs shown in each panel are divided into 6 subsets, as highlighted by different color backgrounds.

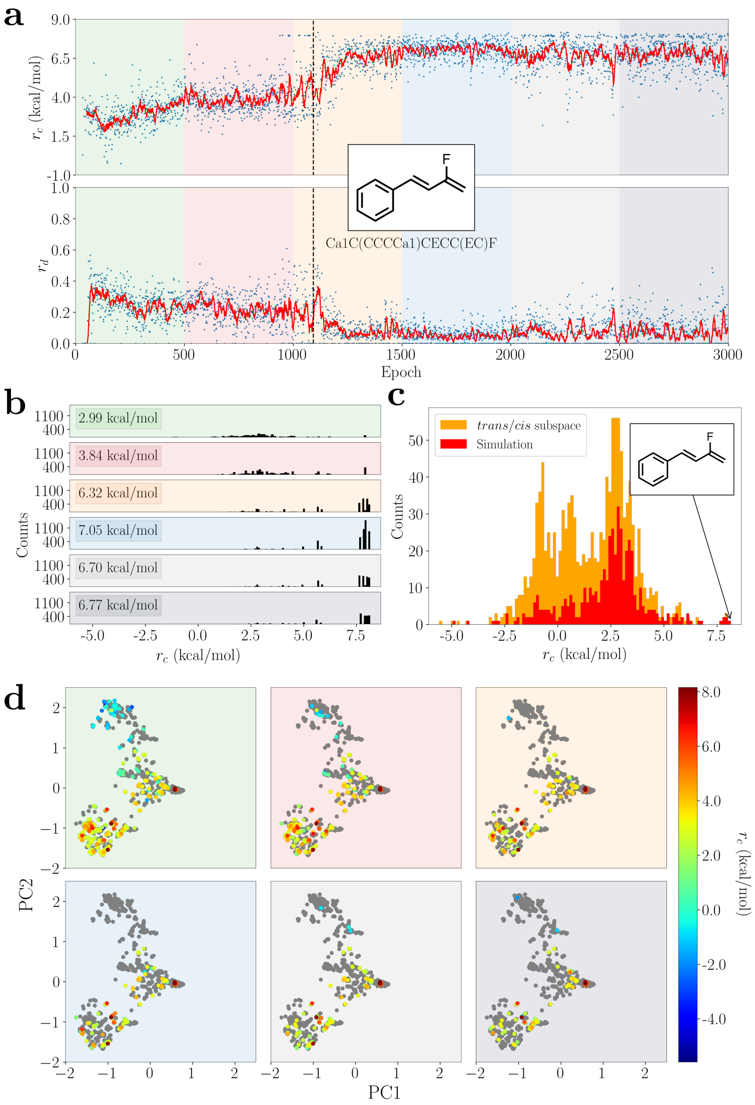

Figure S18: **Inverse design of *trans/cis* isomers with PROTEUS. a**, Time-evolution of the chemical reward during the PROTEUS simulation 10. Both the mean value of each epoch (blue scatter) and the running average (solid red line) are reported. The epoch corresponding to the first generation of the best solution (see the molecular formula in the inset), as ranked in the '*trans/cis* space', is marked with a dashed line. The 3,000 epochs reported are divided into 6 windows with different background colors. **b**, The counts of the generated molecules are plotted as a function of the chemical reward (i.e. the *trans/cis* energy gap), with mean values reported in the insets. **c**, The *trans/cis* energy gap distributions for unique isomers in the '*trans/cis* space' (in orange) and in the PROTEUS simulations (in red) are compared. **d**, For each simulation window, the generated molecules are displayed using the principal components defined for the full E/Z space (and reported in Fig. 1c). The states generated in each window are labeled using the *trans/cis* energy gap values defined by the color bar, while molecules belonging to the *trans/cis* CS that are not explored are reported in grey.

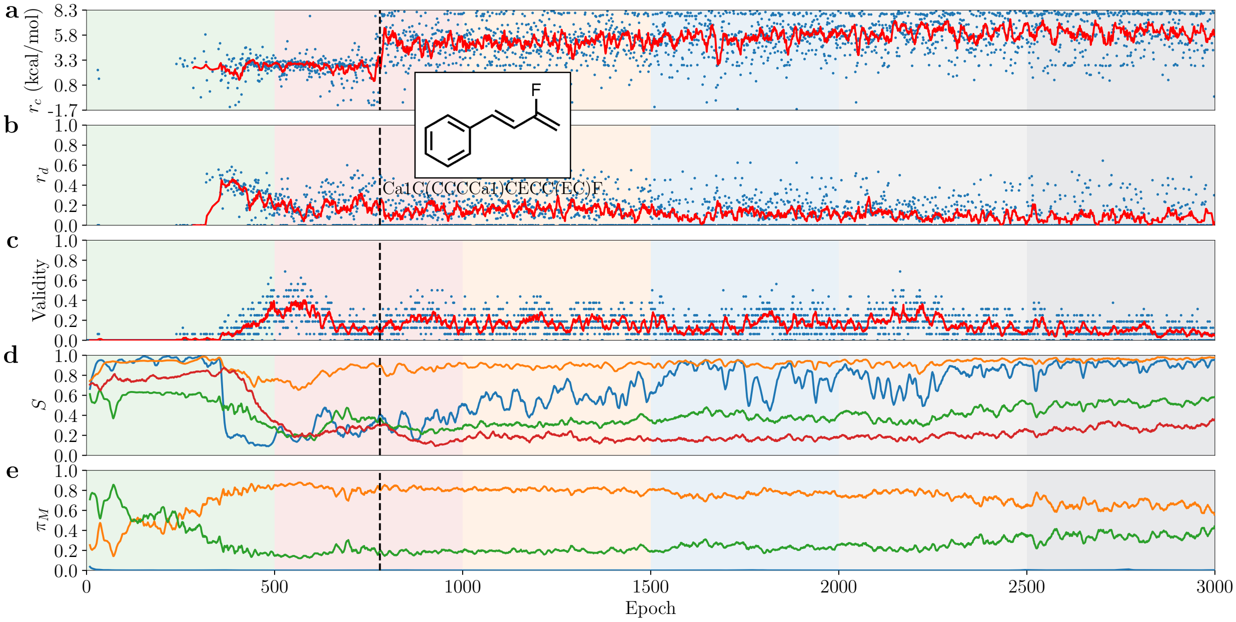

Figure S19: **Simulation 13.** Time-evolution of (**a**) the chemical and (**b**) the diversity reward, and (**c**) the validity density. Both the mean value of each epoch (blue scatter) and the running average (solid red line) are reported. Time-evolving average of the (**d**) entropy value of each policy of the models - M (blue), G^S (yellow), G^D (green), P (red) - and the (**e**) policy value of the master M, `add double-char` (blue), `add single-char` (yellow), `return state` (green). The epoch corresponding to the first generation of the best molecule (inset), as ranked in the 'E/Z space', is marked with a dashed line. The total epochs shown in each panel are divided into 6 subsets, as highlighted by different color backgrounds.

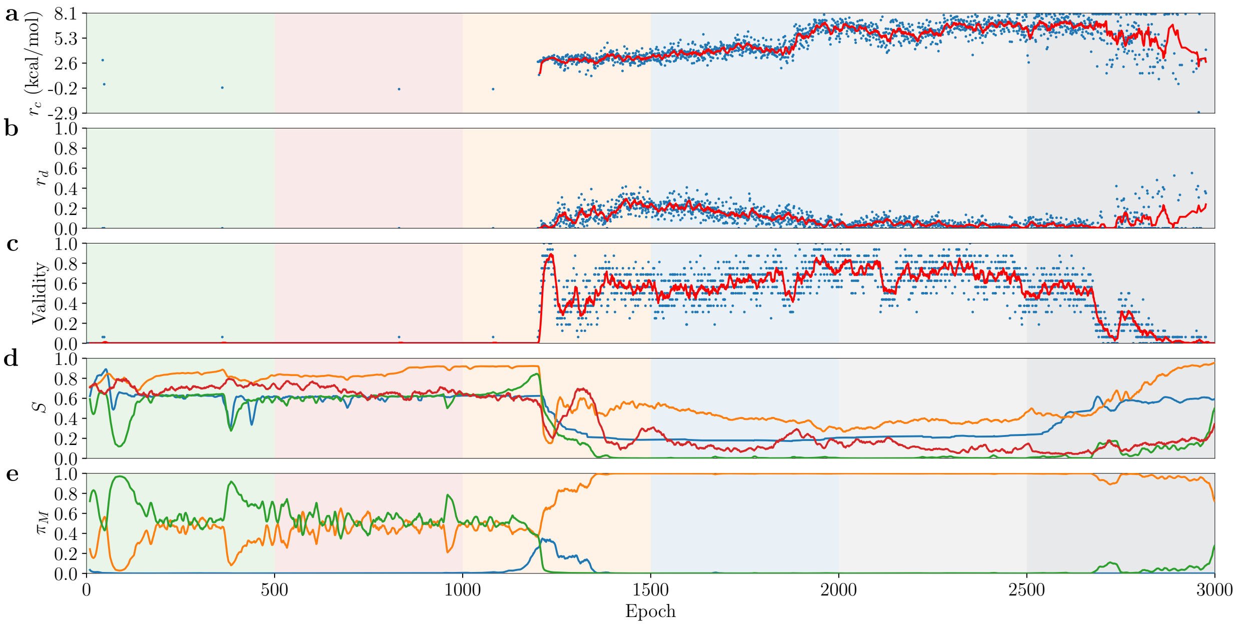

Figure S20: **Simulation 14.** Time-evolution of (**a**) the chemical and (**b**) the diversity reward, and (**c**) the validity density. Both the mean value of each epoch (blue scatter) and the running average (solid red line) are reported. Time-evolving average of the (**d**) entropy value of each policy of the models - M (blue), G^S (yellow), G^D (green), P (red) - and the (**e**) policy value of the master M, `add double-char` (blue), `add single-char` (yellow), `return state` (green). The epoch corresponding to the first generation of the best molecule (inset), as ranked in the 'E/Z space', is marked with a dashed line. The total epochs shown in each panel are divided into 6 subsets, as highlighted by different color backgrounds.

S4.7 Additional results: the *trans*/*cis* 7-token simulation

Along the 7-token simulation, PROTEUS explored 5,762 syntactically valid molecules featuring up to 7 tokens. This subset is composed as follows:

- 4,276 out of 5,762 returned error.
- 1,976 out of 4,276 raised exception due to connectivity inconsistency at the end of the calculation of the r_c.
- 2,185 out of 4,276 raised exception since they are open-shell systems.
- 106 out of 4,276 raised exception during the xTB optimization.
- 9 out of 4,276 raised exception during the DFT optimization.

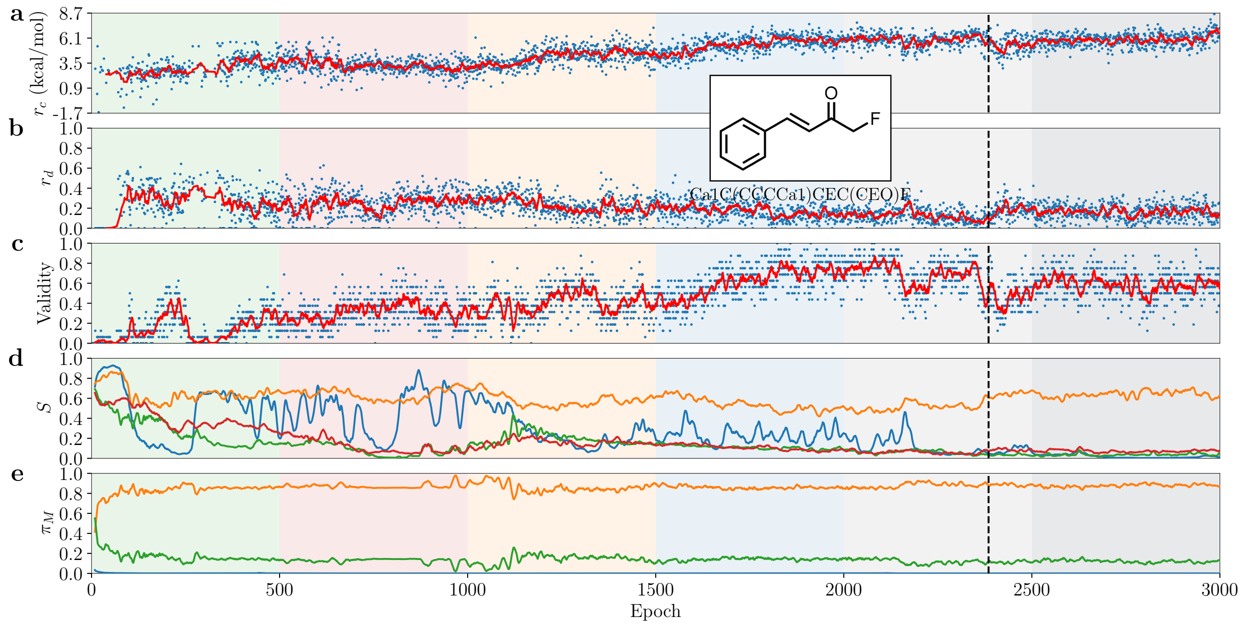

Figure S21: **Simulation 12.** Time-evolution of (**a**) the chemical and (**b**) the diversity reward, and (**c**) the validity density. Both the mean value of each epoch (blue scatter) and the running average (solid red line) are reported. Time-evolving average of the (**d**) entropy value of each policy of the models - M (blue), G^S (yellow), G^D (green), P (red) - and the (**e**) policy value of the master M, `add double-char` (blue), `add single-char` (yellow), `return state` (green). The epoch corresponding to the first generation of the best molecule (inset), as ranked in the 'E/Z space', is marked with a dashed line. The total epochs shown in each panel are divided into 6 subsets, as highlighted by different color backgrounds.

S4.8 Additional results: the 6-token *cis*/*trans* simulation

Inverse designing tailored substituents of the styrene's backbone to maximize the *cis*/*trans* energy gap using 6 P-SMILES tokens is highly challenging. Similarly to the *trans*/*cis* problem, the molecule with the largest energy gap, i.e. `Ca1C(CCCCa1)CECCONZF`, is in a region of low reward values. Moreover, as disclosed by the PCA simulation, `Ca1C(CCCCa1)CECCONZCF` is chemically and structurally more similar to molecules with much smaller energy gaps. In fact, `Ca1C(CCCCa1)CECCONZCF` does not belong to the cluster containing the molecules with positive energy differences, but to one where most of the molecules feature a more stable *cis* conformer than a *trans* one (see the main text). Therefore, PROTEUS must balance extensive exploration of the CS and exploitation of the chemical reward to successfully solve the ID problem.

Fig. S22 summarizes the results of the 6-token *cis/trans* simulation. The overall simulation was 12,000 epochs long, instead of 3,000 as for the other simulations, as expected for a more topologically challenging ID problem as the *cis/trans* one. During the first 4,000 epochs, PROTEUS explores the space of solutions while maximizing r_c. Similarly to what already discussed in the main text, r_c and r_d show opposite trends: the wider the exploitation of the chemical property, the lower the diversity reward (Figg. S22a and S22b). After the ca. 5,000 epochs, PROTEUS prioritizes back the exploration of the chemical space rather than the exploitation of r_c. Such behavior is encouraged by an increment in the total entropy, S, of the master, which pushes PROTEUS to explore new regions of the space of solutions (Fig. S22d). In fact, the r_d increases (Fig. S22b) and the unexplored molecules are generated. Thanks to this second exploration phase, PROTEUS successfully generates `Ca1C(CCCCa1)CECCONZCF` during the 6,734-th epoch. This result is really remarkable, since it proves again that the architecture of PROTEUS promotes the exploration of CS with challenging topologies.

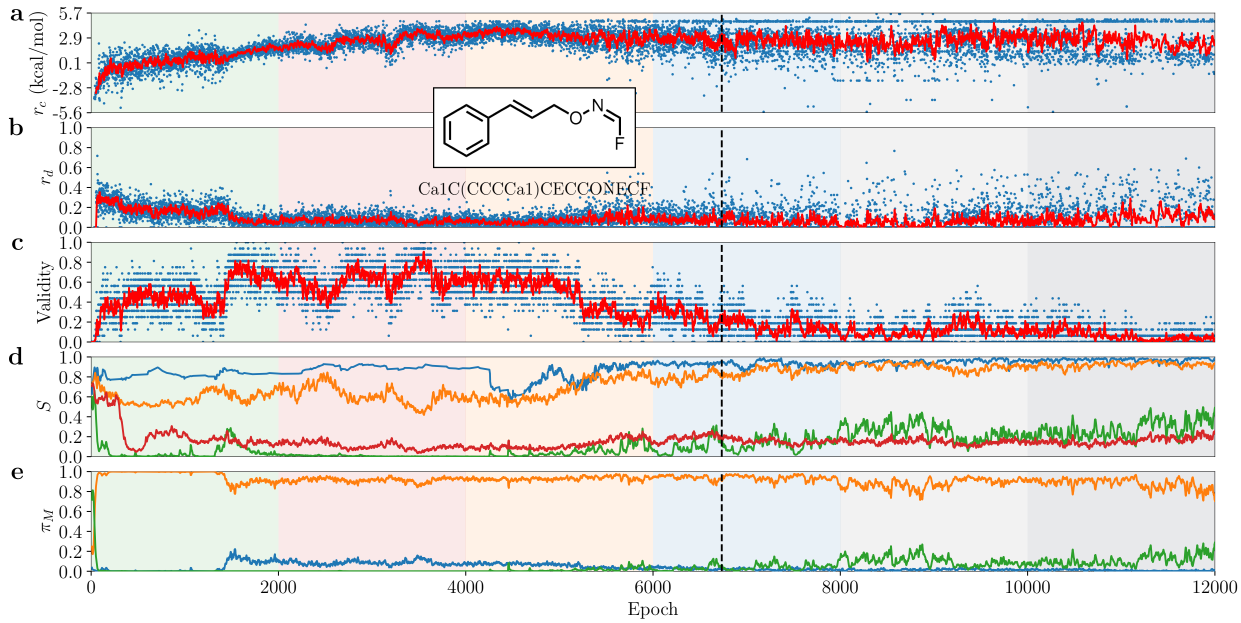

Figure S22: **Simulation 11.** Time-evolution of the **a** chemical and the **b** diversity reward, and the **c** validity density. Both the mean value of each epoch (blue scatter) and the running average (solid red line) are reported. **d** Time-evolving average of the entropy value of each policy of the models: M (blue), G^S (yellow), G^D (green), P (red). **e** Time evolving average policy value of the master M, `add double-char` (blue), `add single-char` (yellow), `return state` (green). The epoch corresponding to the first generation of the best molecule (inset), as ranked in the 'E/Z space', is marked with a dashed line. The total epochs shown in each panel are divided into 6 subsets, as highlighted by different color backgrounds.

S4.9 Sensitivity analysis of exploitation and exploration hyperparameters

The choice of optimal values of the hyperparameters is a key ingredient in RL simulations, even if it must be highlighted that this framework is less influenced by the choice of hyperparameters than other generative techniques.[22]

Among others, α and β govern the exploitation and the exploration of the learning process, respectively, by weighting the r_c and the r_d values in the formulation of the reward r_t. Since the importance of the role played by α and β parameters, we verified how different ratios of those hyperparameters influence the simulations of the 6-token *trans*/*cis* problem. Here, all reamining hyperparameters, as listed in Tab. S4, are kept constant. Results are

given in Tab. S6 and Fig. S23.

When $\alpha : \beta = 1 : 1$, PROTEUS explores the space of solutions broadly while exploiting the task to find the best state (Fig. S23c). During the simulation 43% of the generated states are valid, and 464 are unique states (Tab. S6).

Gratifyingly, when $\beta = 0$, i.e. the r_d contribution is ignored, PROTEUS solves the *trans/cis* problem as well, but exploring a smaller part of the reference chemical space after the same number of epochs, as for the previous simulation. The number of unique valid states generated is 228, while the number of total valid generated states, ρ_v, is 14% of the total states (Tab. S6). The lower exploration is witnessed also by the topological analysis of the explored regions (Fig. S23a) and of the distribution of the reward (Fig. S23b). The comparison of Fig. S23b and Fig. S23d highlights that when the total reward r_t is corrected with the contribution of the chemical diversity, PROTEUS focuses on the broad exploration of the regions related to large reward values.

When $\beta = 2\alpha$ (Figg. S23e and S23f), PROTEUS performs worse than when $\alpha : \beta = 1 : 1$. In fact, the number of unique valid states generated during the simulation drops from 464 to 338 (Tab. S6) and PROTEUS does not generate the best state. The fact that the density of valid solutions lowers is ascribed to an excessive exploration of the chemical space. In fact, the r_d value embeds the structural and chemical diversity between the latest generated states and the previous ones (Methods section). When the reward is doped to explore the most different states possible, PROTEUS tends to generate less meaningful, i.e. more invalid, P-SMILES string.

In summary, this outcome shows how the choice of a proper $\alpha : \beta$ ratio leads to different explorations of the chemical space. Moreover, weighting the r_t with βr_d is a key critical component when exploring chemical spaces with challenging topologies.

Table S6: **Sensitivity analysis.** Influence of the ratio of the hyperparameters α and β on the *trans/cis* simulation. The density of valid states, ρ_v, and the total number of unique valid states are reported.

$\alpha : \beta$	**1 : 0**	**1 : 1**	**1 : 2**
$\rho_v(\%)$	14	43	31
Unique states	228	464	338

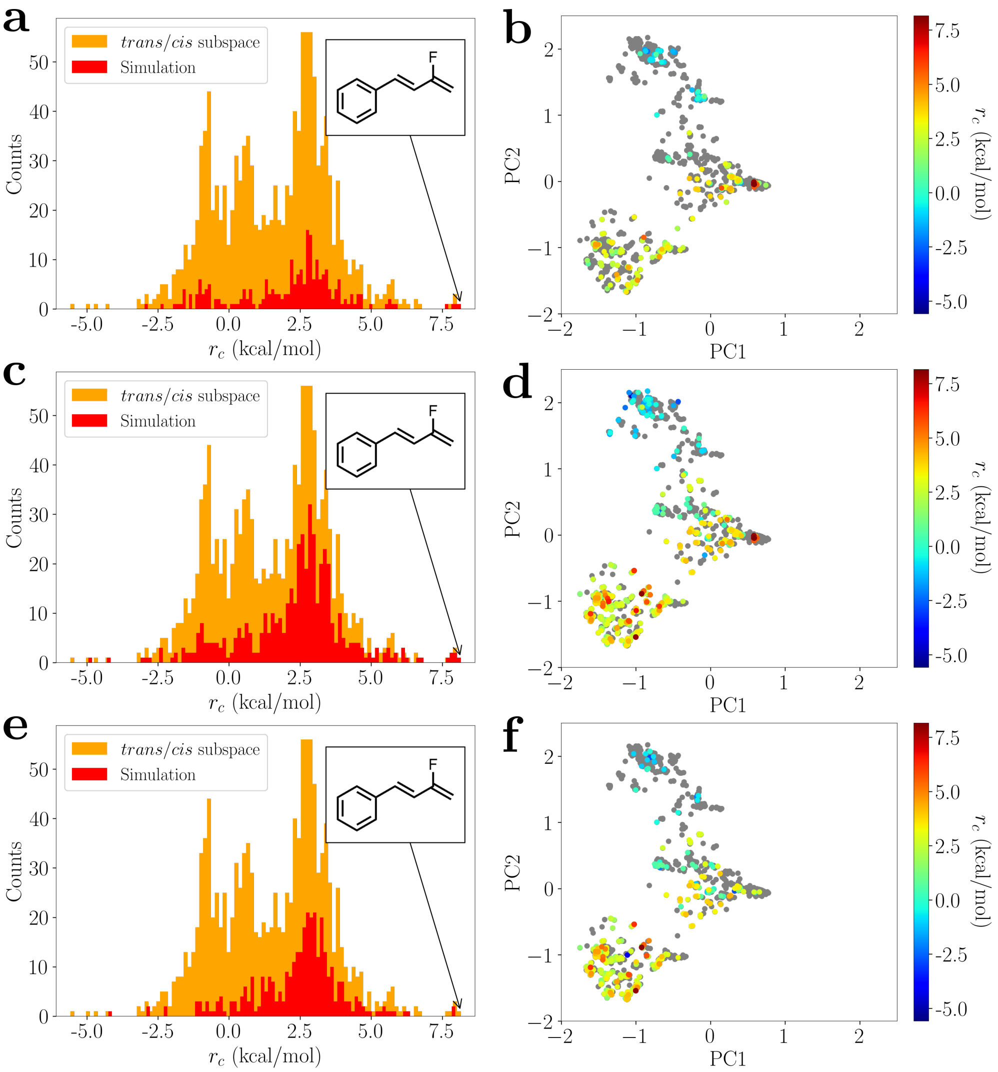

Figure S23: **Sensitivity analysis.** PCA analysis and rewards distributions of the exploration of the chemical space for the *trans/cis* problem according to different $\alpha : \beta$ ratios, i.e., 1:0 (**a** and **b**), 1:1 (**c** and **d**), and 1:2 (**e** and **f**).

References

(1) Krenn, M. et al. SELFIES and the future of molecular string representations. *Patterns* **2022**, *3*, 100588.

(2) Weininger, D. SMILES, a chemical language and information system. 1. Introduction to methodology and encoding rules. *Journal of Chemical Information and Computer Sciences* **1988**, *28*, 31–36.

(3) Weininger, D.; Weininger, A.; Weininger, J. L. SMILES. 2. Algorithm for generation of unique SMILES notation. *Journal of Chemical Information and Computer Sciences* **1989**, *29*, 97–101.

(4) Weininger, D. SMILES. 3. DEPICT. Graphical depiction of chemical structures. *Journal of Chemical Information and Computer Sciences* **1990**, *30*, 237–243.

(5) O'Boyle, N. M.; Banck, M.; James, C. A.; Morley, C.; Vandermeersch, T.; Hutchison, G. R. Open Babel: An open chemical toolbox. *Journal of Cheminformatics* **2011**, *3*, 1–14.

(6) O'Boyle, N. M.; Morley, C.; Hutchison, G. R. Pybel: a Python wrapper for the OpenBabel cheminformatics toolkit. *Chemistry Central Journal* **2008**, *2*, 1–7.

(7) Vaswani, A.; Shazeer, N.; Parmar, N.; Uszkoreit, J.; Jones, L.; Gomez, A. N.; Kaiser, L. u.; Polosukhin, I. Attention is all you need. *Advances in Neural Information Processing Systems* **2017**,

(8) Schulman, J.; Wolski, F.; Dhariwal, P.; Radford, A.; Klimov, O. Proximal Policy Optimization Algorithms. 2017; arXiv:1707.06347 [cs.LG].

(9) Frisch, M. J.; Pople, J. A.; Binkley, J. S. Self-consistent molecular orbital methods 25. Supplementary functions for Gaussian basis sets. *The Journal of Chemical Physics* **1984**, *80*, 3265–3269.

(10) Clark, T.; Chandrasekhar, J.; Spitznagel, G. W.; Schleyer, P. V. R. Efficient diffuse function-augmented basis sets for anion calculations. III. The 3-21+G basis set for first-row elements, Li–F. *Journal of Computational Chemistry* **1983**, *4*, 294–301.

(11) McLean, A. D.; Chandler, G. S. Contracted Gaussian basis sets for molecular calculations. I. Second row atoms, Z=11–18. *The Journal of Chemical Physics* **1980**, *72*, 5639–5648.

(12) Krishnan, R.; Binkley, J. S.; Seeger, R.; Pople, J. A. Self-consistent molecular orbital methods. XX. A basis set for correlated wave functions. *The Journal of Chemical Physics* **1980**, *72*, 650–654.

(13) Grimme, S.; Antony, J.; Ehrlich, S.; Krieg, H. A consistent and accurate ab initio parametrization of density functional dispersion correction (DFT-D) for the 94 elements H-Pu. *The Journal of Chemical Physics* **2010**, *132*, 154104.

(14) Grimme, S.; Ehrlich, S.; Goerigk, L. Effect of the damping function in dispersion corrected density functional theory. *Journal of Computational Chemistry* **2011**, *32*, 1456–1465.

(15) Marenich, A. V.; Cramer, C. J.; Truhlar, D. G. Universal solvation model based on solute electron density and on a continuum model of the solvent defined by the bulk dielectric constant and atomic surface tensions. *The Journal of Physical Chemistry B* **2009**, *113*, 6378–6396.

(16) Landrum, G. Rdkit documentation. *Release* **2013**, *1*, 4.

(17) Jolliffe, I. T.; Cadima, J. Principal component analysis: a review and recent developments. *Philosophical Transactions of the Royal Society A: Mathematical, Physical and Engineering Sciences* **2016**, *374*, 20150202.

(18) Van der Maaten, L.; Hinton, G. Visualizing data using t-SNE. *Journal of Machine Learning Research* **2008**, *9*.

(19) Pedregosa, F. et al. Scikit-learn: Machine Learning in Python. *Journal of Machine Learning Research* **2011**, *12*, 2825–2830.

(20) McInnes, L.; Healy, J.; Melville, J. Umap: Uniform manifold approximation and projection for dimension reduction. *arXiv preprint arXiv:1802.03426* **2018**,

(21) McInnes, L.; Healy, J.; Saul, N.; Grossberger, L. UMAP: Uniform Manifold Approximation and Projection. *The Journal of Open Source Software* **2018**, *3*, 861.

(22) Anstine, D. M.; Isayev, O. Generative Models as an Emerging Paradigm in the Chemical Sciences. *Journal of the American Chemical Society* **2023**, *145*, 8736–8750.
